# Adaptive deep brain stimulation for dynamic gait control in Parkinson’s disease: a randomized feasibility trial

**DOI:** 10.1038/s41591-026-04434-2

**Published:** 2026-06-15

**Authors:** Kenneth H. Louie, Jannine P. Balakid, Jessica E. Bath, Seongmi Song, Hamid Fekri Azgomi, Jacob H. Marks, Julia T. Choi, Philip A. Starr, Doris D. Wang

**Affiliations:** 1 Department of Neurological Surgery, University California, San Francisco, San Francisco, CA USA; 2 Department of Physical Therapy and Rehabilitation Sciences, University of California, San Francisco, San Francisco, CA USA; 3 Department of Applied Physiology & Kinesiology, University of Florida, Gainesville, FL USA; 4 Graduate Program in Bioengineering, University of California, Berkeley and University of California, San Francisco, San Francisco, CA USA; 5 Weill Institute for Neurosciences, University of California, San Francisco, San Francisco, CA USA

**Keywords:** Translational research, Parkinson's disease, Biomarkers, Biomedical engineering

## Abstract

A randomized crossover study of five patients with Parkinson’s disease (PD) demonstrates that gait-synchronized adaptive deep brain stimulation is feasible and safe, and reduces falls compared with continuous stimulation. Gait dysfunction in PD is a major source of disability and is often insufficiently treated by continuous deep brain stimulation (cDBS). Although adaptive DBS (aDBS) has shown efficacy for other motor symptoms using β-based, state-driven neural signals, gait is a dynamic, cyclical behavior that may require temporally precise modulation. Here we evaluated a behavior-contingent aDBS approach that synchronizes stimulation to gait phase. We reported a single-center, blinded, randomized, crossover study evaluating the feasibility of identifying patient-specific biomarkers to drive aDBS. The primary outcome was feasibility of successful identification of gait-phase biomarkers to implement aDBS. Five participants with PD undergoing pallidal DBS and subdural electrode paddle implantation were enrolled. We successfully identified personalized gait-phase biomarkers from cortical or pallidal field potentials in all five patients and embedded them into a bidirectional neurostimulator. During acute in-clinic testing, aDBS improved step variability and step symmetry versus cDBS. Three participants subsequently completed a double-blinded, multi-day crossover phase. In this setting, aDBS maintained general motor symptom control, reduced falls and yielded patient-specific gait improvements. No adverse events occurred and aDBS was well tolerated. These findings establish the feasibility of biomarker-driven, movement-synchronized neuromodulation and support the development of a larger randomized trial to determine clinical efficacy. ClinicalTrial.gov registration: NCT04675398.

## Abstract (web summary)

A randomized crossover study shows that gait-phase-synchronized adaptive deep brain stimulation is feasible and safe, and reduces falls compared to continuous stimulation in Parkinson’s disease.

## Main

Gait disturbances in people with Parkinson’s disease (PD) are among the most debilitating symptoms, reducing quality of life, increasing the risk of falls and diminishing independence^1–3^. Although deep brain stimulation (DBS) therapy may initially improve gait disturbances, its efficacy often diminishes over time^4–10^. Previous attempts to enhance gait outcomes with DBS, such as lowering stimulation frequency^11–18^ or changing stimulation contact location^19–21^, have produced mixed results. Improvements in gait often come at the cost of worsened tremor control^18,22^ or a reduction in falls accompanied by increased gait variability^23^. Thus, a new stimulation approach is needed to improve gait without compromising the treatment of other PD symptoms.

Current DBS approaches may fail to address gait disturbances because gait is a complex and dynamic motor behavior requiring the precise coordination of muscle contractions and relaxations across all limbs. During locomotion, the human body must maintain balance while each leg alternates between the stance phase, when the foot contacts the ground, and the swing phase, when the foot is in the air. However, current DBS therapy typically delivers continuous stimulation without adjusting to patients’ movement states, which can exacerbate gait impairments^24,25^.

Recent studies have demonstrated that local field potential (LFP) spectral power within deep brain nuclei fluctuates naturally throughout the day, influenced by sleep^26^, mood^27^ and medication states^28–30^. Although most of these electrophysiological insights derive from the subthalamic nucleus (STN), parallel dynamics exist in the internal globus pallidus (GPi). Without accounting for these fluctuating neural dynamics, continuous stimulation of the STN or GPi may lead to suboptimal or adverse motor effect. For instance, during ‘on’ medication states, when dopaminergic therapy effectively controls motor symptoms, continuous DBS (cDBS) can cause stimulation-induced dyskinesia^28,31–34^. From a network perspective, although neural entrainment to stimulation is often associated with improved motor performance, continuous stimulation may impose a persistently entrained, temporally invariant neural state^29,35,36^, limiting the capacity of corticobasal ganglia circuits to dynamically reconfigure in accordance with ongoing behavioral demands^37–43^.

Adaptive DBS (aDBS), which adjusts stimulation parameters based on real-time neural signals, has emerged as a promising approach to address changes in physiological demand^44,45^. Most current aDBS implementations rely on relatively slow-varying, tonic neural features, most commonly β-band activity, that reflect global motor or medication state. These state-adaptive strategies are effective for improving ‘on’ times without bothersome dyskinesia^46–50^, but are not inherently aligned with the rapid temporal structure of dynamic motor behaviors such as gait.

**Fig. 1:**
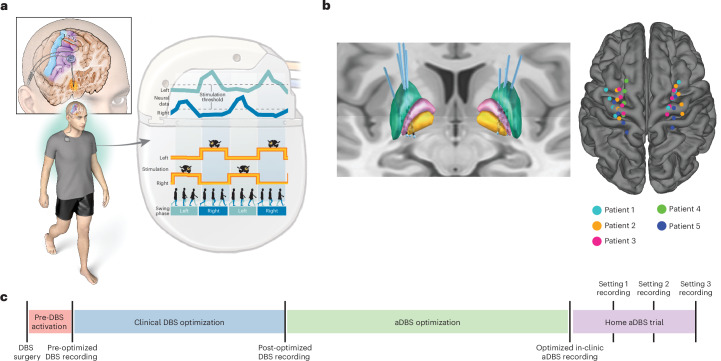
Overview of aDBS system and study design. **a**, An aDBS control strategy for independent, phase-specific modulation of stimulation amplitude in each hemisphere. Neural signals were continuously recorded from the internal segment of the GPi or cortex (teal and blue traces) and spectral power within a specified frequency band was computed. When spectral power crossed a defined threshold (dashed gray line), stimulation amplitude (yellow trace) increased to the clinically optimized value during contralateral leg swing and decreased to 0.5× the optimized amplitude during other gait phases. **b**, Reconstruction of DBS leads and cortical electrode strips, normalized to Montreal Neurological Institute space. Cortical electrodes were overlaid on a standard brain atlas. The internal pallidum is shown in orange, external pallidum in pink and striatum in green. **c**, Study timeline divided into four phases: phase 1: postoperative period before DBS activation (red); phase 2: standard clinical optimization of DBS parameters (blue); phase 3: testing and optimization of adaptive settings (green); and phase 4: home testing of optimized aDBS settings in three patients (patients 2–4) under double-blind conditions. Three stimulation settings were tested: aDBS ramp-up (0.5× to 1.0× amplitude) during contralateral leg swing, aDBS ramp-down (1.0× to 0.5× amplitude) during contralateral leg swing and clinically optimized cDBS.

Here we introduce a behavior-contingent aDBS paradigm that operates on a fundamentally different principle. Rather than modulating stimulation based on global motor state, this approach synchronizes stimulation to discrete phases of the gait cycle, enabling subsecond, movement-phase-locked modulation of neural activity (Fig. 1a). By aligning stimulation delivery with the temporal structure of locomotion, this strategy is designed to complement existing state-adaptive approaches and specifically target dynamic motor impairments. We investigated the pallidum and motor cortical areas for evidence of gait-phase modulation and developed a data-driven approach to identify patient-specific biomarkers of contralateral leg swing in each hemisphere. During free overground walking, patients exhibited unique frequency bands and recording sites that captured spectral power changes during contralateral swing. Acute in-clinic application of aDBS reduced step-length and step-time variability and improved gait symmetry compared to clinically optimized cDBS. Importantly, in double-blinded trial testing of cDBS and two different gait-phase synchronized aDBS settings over the longer term at home, patients not only tolerated rapid stimulation adjustments, but also maintained general PD symptom control and exhibited multiple improved gait metrics. Together, these findings demonstrate the feasibility of rapid, gait-phase-synchronized aDBS for improving gait in PD, a promising new therapy for treating advanced gait disorders in PD.

## Results

### Patient overview and study design

Patients were recruited from a cohort of individuals with PD and gait problems who were eligible for conventional DBS therapy. Patients 1–4 (Table 1) were enrolled in a study using an investigational bidirectional neural stimulator^49^ (Summit RC + S, model B35300R, Medtronic, Inc.) to develop and test aDBS therapies targeting motor and gait functions (ClinicalTrials.gov ID: NCT04675398; Fig. 1a). Patient 5 crossed over from a parallel trial (ClinicalTrials.gov ID: NCT03582891) using the same device.

**Table 1:** Patient demographics

Demographics	Patient 1	Patient 2	Patient 3	Patient 4	Patient 5
Sex/age (years)	M/68	M/64	M/68	F/68	M/53
Disease duration (years)	21	12	8	4	18
LEDD^a^ (mg)	2,590	1,514	1,025	618	1,353
**Clinical measurements**					
MDS-UPDRS-III OFF/ON^b^	37/16	42/19	31/17	21/9	39/16
MDS-UPDRS-III PIGD subscore OFF/ON^c^	−/−	10/3	3/2	2/2	1/0
MiniBEST LOW^d^/ON	13/17	17/25	22/21	19/21	−/−

^a^Levodopa equivalent daily dose.

^b^Rigidity and postural stability scores excluded due to remote video examination.

^c^Total of UPDRS-III items: 3.9–3.11 and 3.13.

^d^LOW, low medication state defined as the time period between the end of their current medication cycle to the next. Achieved by delaying next dose during testing.

Note: ‘−/−‘ indicates that the data could not be acquired due to remote assessment restrictions (patient 1) or safety concerns (patient 5).

Patients underwent DBS surgery with implantation of a quadripolar depth electrode (Medtronic, model 3387) targeting the internal segment of the GPi and a quadripolar subdural electrocorticography paddle (Medtronic, model 0913025) placed over the premotor (PM) and primary motor (M1) cortices (patients 1–4) or over the sensorimotor cortices (M1 and S1) in patient 5. Electrodes were implanted bilaterally in patients 1–3 and 5 and unilaterally in patient 4 due to predominantly unilateral tremor and leg dragging (Fig. 1b). Each Summit RC + S device was connected to a DBS electrode and a subdural cortical paddle from the same brain hemisphere. LFPs and gait kinematic data were recorded during overground walking at self-selected speeds for at least 250 steps, before stimulation was activated (26–43 d postoperatively), after DBS parameter optimization according to standard clinical care (140–520 d postoperatively) and during aDBS parameter optimization (4–10 sessions). LFPs were recorded from pallidal contacts adjacent to the stimulating electrode and from cortical contacts (PM and M1 or M1 and S1) using adjacent electrode pairs.

Using patient-specific neural data, we developed an aDBS therapy that modulated stimulation amplitude between 0.5× and 1.0× the clinically optimized setting during contralateral leg swing. Control signals were derived from single-frequency band power at one recording site per implanted hemisphere, identified through a data-driven search. Candidate neural features were evaluated based on their ability to distinguish contralateral leg swing from other gait phases. Optimal neural features were embedded into the Summit RC + S device for each implanted hemisphere (bilaterally for patients 1–3 and 5 and unilaterally for patient 4) and adaptive parameters were manually refined to maximize detection accuracy.

For three patients (patients 2, 3 and 4), a double-blind crossover experiment was conducted, randomizing three stimulation conditions: cDBS; aDBS ramping from 0.5× to 1.0× their clinically optimized amplitude (ramp-up aDBS (RU-aDBS)) and aDBS ramping from 1.0× to 0.5× the clinically optimized amplitude during contralateral leg swing (ramp-down aDBS (RD-aDBS)). Each condition was applied continuously for 8–10 d, during which patients filled out a daily motor diary where they rated nongait-related PD symptoms and documented their number of falls and freezing episodes (Fig. 1c). In addition, patients wore a triaxial accelerometer (Rover, Sensoplex Inc.) that passively collected data, which were then uploaded to the manufacturer’s cloud platform for data processing and quantification of at-home gait metrics.

### Primary feasibility outcome: neural biomarkers of leg swing during overground walking

**Fig. 2:**
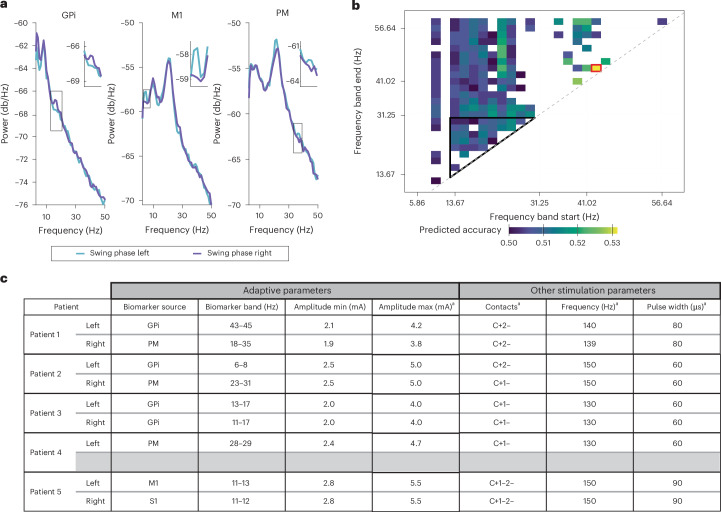
Identification of gait-phase-specific neural biomarkers using cortical and subcortical recordings. **a**, Example power spectral density plots of average values during left and right swing phase in the GPi (left hemisphere), M1 (right hemisphere) and PM (right hemisphere) areas. Detectable power differences are observed, indicating each area’s potential to find a candidate gait-phase biomarker. Not all patients exhibited detectable power differences in all recording locations. Individual variations between canonical frequency bands are provided in Extended Data Fig. 1. **b**, Example brute-force search results from patient 1’s left hemisphere for contralateral swing-phase-specific frequency bands using Summit RC + S power calculations. Bands achieving at least 50% accuracy are colored and bands with <50% accuracy are omitted. Many bands within the β range (outlined by a black triangle) were identified, although the optimal band (42–44 Hz) was outside canonical β and is highlighted in red. **c**, Table of patient-specific biomarkers to detect contralateral leg swing phase. ^a^Clinically optimized parameters according to standard clinical care. Min, minimum; Max, maximum.

**Extended Data Fig. 1:**
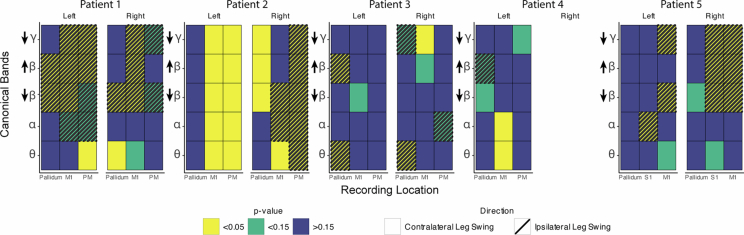
Differences in canonical frequency band powers in distinguishing leg swings in individual patients. Heatmap of canonical band significance test for distinguishing between ipsilateral or contralateral leg swing phases compared to the entire walking period. Color indicates significance level: p<0.05 (yellow), p<0.15 (green), p>0.15(blue). Frequency bands: Θ = 4-7 Hz, α = 7-12 Hz, ↓β = 13-20 Hz, ↑ β = 20-30 Hz, ↓γ = 30–50 Hz. Recording location: Pallidum = GPi, M1 = primary motor cortex, PM = premotor cortex, S1 = primary somatosensory cortex.

We investigated neural dynamics during natural overground gait by examining LFPs recorded from the pallidum and PM, M1 and S1. Individual gait cycles were extracted offline and fast Fourier transform (FFT) spectral power was calculated in the following canonical frequency bands—θ (4–7 Hz), α (7–12 Hz), low β (13–20 Hz), high β (20–30 Hz) and low γ (30–50 Hz) bands—to assess gait phase-dependent modulation. Substantial modulation was observed for both ipsilateral and contralateral swing phases in the pallidum and cortical areas, with individual differences in the frequency bands achieving significance (Fig. 2a and Extended Data Fig. 1). Across patients, M1 exhibited the highest proportion of hemispheres with at least one discriminative canonical frequency band (eight of nine), followed by the pallidum (five of nine), PM (three of nine) and S1 (two of nine) (Extended Data Fig. 1). In two patients (patients 4 and 5), pallidal recordings did not result in any frequency bands capable of distinguishing swing phases, whereas patient 2 exhibited a discriminative band only in the right hemisphere.

Given the observed interpatient and intrapatient variability, we next implemented a data-driven approach to identify patient-specific biomarkers of contralateral swing phase that has higher discriminatory power. This approach involved a grid search across all recording locations, evaluating all possible frequency band combinations between 2.5 Hz and 50 Hz and quantifying contralateral swing-phase decoding accuracy (Fig. 2b). Compared to canonical bands, these individualized frequency bands revealed subfrequency ranges that better distinguished contralateral swing phase and, in most cases, achieved higher decoding accuracy (Fig. 2b, red square). The origin of patient-specific biomarkers differed among patients and even between hemispheres of the same patient. Optimal gait-phase biomarkers were identified from the pallidum in four hemispheres, the PM in three hemispheres and M1 and S1 with one hemisphere each (Fig. 2c).

### Primary feasibility outcome: accuracy and stability of contralateral leg swing biomarkers in real-time aDBS control

Patient-specific contralateral swing-phase biomarkers were embedded in each patient’s neural stimulator using the on-device linear discriminant analysis classifier, after which adaptive parameters were optimized. Two parameters were critical: ramping rate and amplifier blanking. The ramping rate, which determines the speed of stimulation amplitude changes after a state transition, was titrated for each patient from 1.9 mA per 400 ms upward until either side effects emerged or biomarker accuracy declined due to rapid amplitude changes. Amplifier blanking, which is the brief deactivation of the onboard recording mechanism^51^, was set to 1 ms, which was sufficient to minimize ramping-related artifacts.

**Fig. 3:**
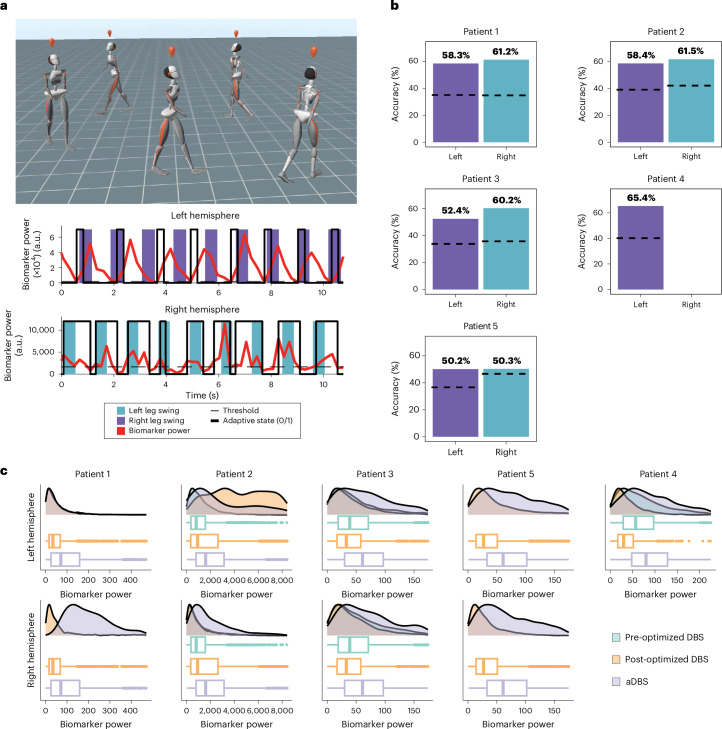
Real-time performance and stability of rapid gait-phase aDBS. **a**, Example of aDBS implementation during overground walking at a self-selected speed. The spectral power of the selected biomarker (red trace), determined through a data-driven approach, increased above the threshold (dashed black line) during contralateral leg swing (purple, left; teal, right) and decreased below the threshold during other gait phases. When spectral power exceeded the threshold, the DBS system switched to ‘state 1’, increasing stimulation amplitude (solid black line); when power fell below the threshold, the system returned to ‘state 0’, decreasing stimulation amplitude. **b**, Accuracy of optimized biomarkers across patients exceeding chance level, with an average improvement of 18.6%. Accuracy was defined using equation (2) as the ratio of the Summit RC + S state duration in true positives (in contralateral swing phase) plus the duration in true negatives (not in contralateral swing phase) to the total recording duration. Chance level (dashed black line) was determined by the mean proportion of time spent in contralateral leg swing. **c**, Scaled kernel density estimates (normalized to a peak of one) and their associated box plots of biomarker distributions across pre-optimized DBS, post-optimized DBS and aDBS conditions in the left (top) and right (bottom) hemispheres. Data presented as box plots follow this format: the center line is the median, the box bounds are the 25th and 75th percentiles and the whiskers are 1.5× the IQR; individual points are outliers (minima and maxima). Outliers, defined as values beyond 1.5× the IQR, were excluded from statistical testing but are shown to visualize distribution extremes. The magnitude of distribution shifts across conditions was quantified using Cohen’s *d* effect size. Sample sizes (*n*), Cohen’s *d* values and 95% CIs (estimated via bootstrapping, 1,000 iterations) are detailed in Supplementary Table 1.

In real-time testing, the stimulators accurately detected subsecond gait-phase transitions: during contralateral leg swing, the binary classifier switched from state 0 to state 1, returning to state 0 during other gait phases (Fig. 3a and Supplementary Video 1). The stimulator is able to rapidly ramp up and down stimulation amplitude from 0.5× to 1.0× of the patient’s clinically optimized amplitude during contralateral leg swing. Detection errors occasionally occurred, resulting in shortened or prolonged periods in state 1. False positives during nongait activities (for example, sitting with arm or leg movement) were infrequent and not time locked to specific movements (Supplementary Videos 2 and 3). Across patients, stimulation amplitude was significantly increased during contralateral leg swing relative to chance, defined as the proportion of the gait cycle spent in swing phase, with an average increase of 18.6% (Fig. 3b).

Biomarker stability was assessed by comparing the spectral power of each patient’s gait-phase-specific frequency band across clinical DBS optimization and aDBS testing phases and evaluating the differences in power value distribution. Significant differences in aDBS biomarker power were observed between stimulation conditions in all patients except for the left hemisphere of patient 1: degrees of freedom (d.f.) = 1 for patients 1 and 5, d.f. = 2 for patients 2–4, *n* = 1,255–5,254, *H*(d.f.) = 0.56–1,091, all significant *P* < 0.0001, nonsignificant *P* = 0.45 (Fig. 3c). However, effect-size analysis revealed mostly negligible-to-moderate differences: among 19 comparisons, 2 were negligible, 5 were small, 7 were moderate and 5 were large^52^. Negligible-to-small differences (Cohen’s *d* = −0.038 to +0.428, 95% confidence interval (CI) = −0.57 to +0.36) corresponded to an 83.4–98.4% overlap in power values^53^. Moderate differences (Cohen’s *d* = −0.591 to +0.724, 95% CI = −0.84 to +0.81) corresponded to a 71.9–76.8% overlap. Large effects were observed in four patients but were limited to one hemisphere each. Overall, patient biomarker spectral power varied modestly across timepoints, with a high degree of overlap between distributions.

### Primary clinical outcome: gait parameter changes—in-clinic aDBS versus cDBS

The acute effects of aDBS on gait were assessed against each patient’s clinically optimized cDBS setting. Patients arrived on cDBS and were then switched to individualized aDBS parameters, in which stimulation amplitude ramped from 0.5× to 1.0× the clinically optimized value during the swing phase. After 12–15 min on aDBS, patients completed 250 overground steps across 2 recordings.

**Fig. 4:**
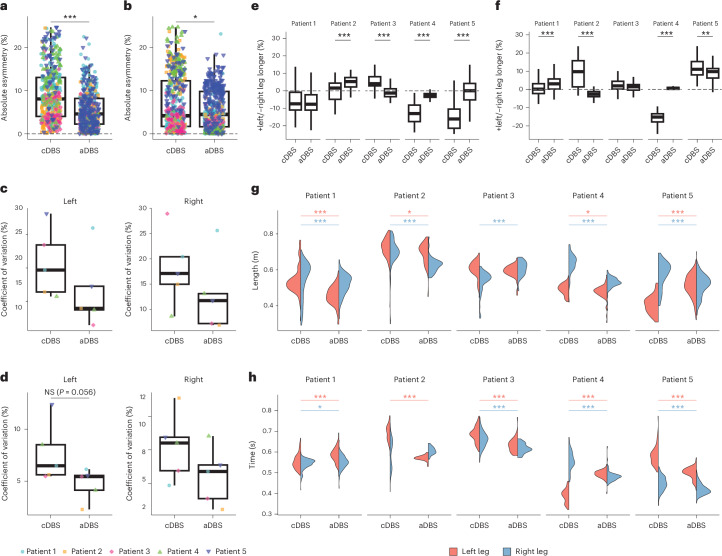
Gait metric changes with aDBS compared to clinically optimized DBS. **a–d**, Group-level analysis of absolute step-length asymmetry (**a**), step-time asymmetry (**b**), step-length variability (**c**) and step-time variability (**d**). **e**,**f**, Individual patient-level analysis of step-length (**e**) and step-time (**f**) symmetry. Values above 0 (positive) mean the left step length is longer; values below 0 (negative) mean the right step is longer. **g**,**h**, Density distributions of individual step length (**g**) and step time (**h**) for left and right legs. Data are presented as box plots (**a**–**f**) and violin plots (**g** and **h**). Data presented as box plots follow this format: the center line is the median, the box bounds the 25th and 75th percentiles and the whiskers 1.5× the IQR. Patients are indicated by unique symbols (patient 1: circle; patient 2: square; patient 3: diamond; patient 4: up-triangle; and patient 5: down-triangle). Gait metrics represent repeated measures nested within *n* = 5 patients. Comparisons were performed using a two-sided Wilcoxon’s rank-sum test. Outliers, defined as values beyond 1.5× the IQR, were excluded. Sample sizes, Wilcoxon’s test statistics and *P* values are detailed in Supplementary Table 2. *P < 0.05; **P < 0.01; ***P < 0.001; NS, not significant.

Group-level analyses indicated improved gait symmetry under aDBS compared to cDBS. Wilcoxon’s rank-sum tests identified significant differences in step-length asymmetry (*n* = 383, versus *n* = 423, Wilcoxon’s *W* = 109,982, *P* < 0.001) and step-time asymmetry (*n* = 390 versus *n* = 476, *W* = 100,426, *P* < 0.05). Median step-length asymmetry showed a 3.5% decrease and a 3.1% reduction in interquartile range (IQR). In contrast, median step-time asymmetry changed minimally (+0.29%), but showed a 2.4% reduction in IQR (Fig. 4a,b). Analysis of step metrics variability showed bilateral reductions in median step-length variability of 39.1% (left) and 31.6% (right) and step-time variability reductions of 16.0% (left) and 26.6% (right), although these changes did not reach significance: *n* = 5, *W* = 18–22, *P* = 0.056–0.310 (Fig. 4c,d).

On an individual level, patient responses varied from one another. Three patients demonstrated significant improvements in step-length symmetry, shifting medians toward 0%, indicative of more balanced left–right step lengths and reducing variability by an average of 2.9% (*W* = 958–2,067, *P* < 0.0001; Fig. 4e). Patient 2 exhibited worsened median symmetry (+1% to +5%) but improved variability by 3.9%, whereas patient 1 showed no significant change. For step-time symmetry, three patients improved significantly (*W* = 0–7,140, *P* < 0.0001), patient 1 worsened due to prolonged left-leg step time and patient 3 showed a small, nonsignificant improvement (−0.47%, *P* = 0.132) (Fig. 4f). Step-length variability decreased bilaterally in 3 patients and unilaterally in 1, with an average reduction of 60.8% (left) and 78.4% (right), whereas step-time variability decreased bilaterally in 4 patients by 62.9% (left) and 55.6% (right). As with step-length variability, patient 1 exhibited bilateral increases in both step-length (+30.6%) and step-time variability (+26.6%). Notably, this lack of response corresponded to a distinct clinical profile. Patient 1 experienced chronic instability of effective cDBS settings, manifesting as severe dyskinesia, and required the longest duration of standard clinical optimization (520 d).

Despite these symmetry and variability improvements, absolute step-length and step-time changes varied between limbs. Significant bilateral step length decreases (*W* = 2,495–15,123, *P* < 0.05) occurred in three patients (patients 1, 2 and 4), whereas two showed unilateral decreases (patients 3 and 5) (Fig. 4g). Decreases ranged from 1% to 16%, whereas patients 3 and 5 exhibited large unilateral increases (13% and 26%, respectively). Step-time changes did not consistently mirror step-length changes. Patients 2 and 4 displayed mixed step-time responses with opposite limb directionality, whereas patients 3 and 5 showed step-time decreases accompanying step-length increases (Fig. 4h).

### Secondary clinical outcome: double-blinded crossover trial shows aDBS decreased falls compared to cDBS

To understand the impact of chronic aDBS stimulation on gait and other PD symptoms, we conducted a double-blind, multi-day evaluation comparing two aDBS strategies—RU-aDBS, previously tested acutely, and a new RD-aDBS paradigm in which stimulation amplitude decreased from 1.0× to 0.5× during contralateral leg swing—against each patient’s cDBS setting (Supplementary Video 4). We hypothesized that ramp-up stimulation during contralateral leg swing may suppress excessive pathological oscillations whereas the ramp-down simulation may permit dynamic, physiological oscillations during this phase of the gait cycle. Three patients (patients 2, 3 and 4) completed this portion of the study. Patient 1 had undergone an abbreviated test of at-home aDBS due to the logistics of living far from our site. Patient 5 had significant disease progression and became wheelchair bound before long-term aDBS testing.

**Fig. 5:**
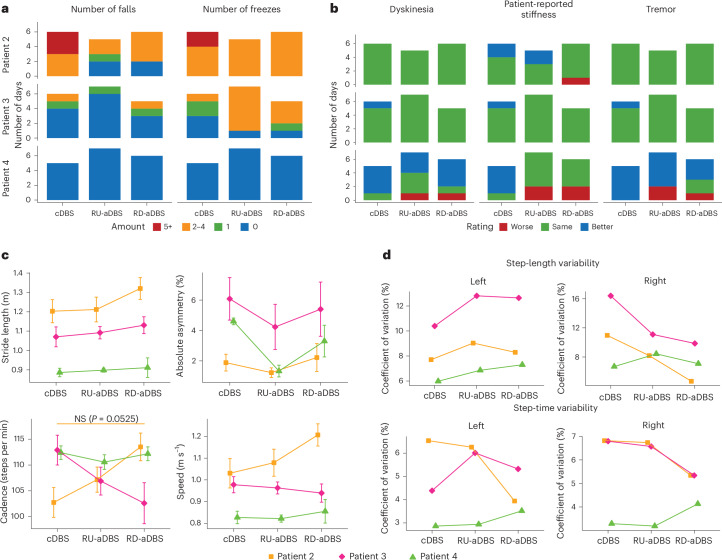
Long-term at-home gait metric changes in a randomized double-blinded comparison of cDBS compared to two aDBS settings. Stimulation settings are consistent across all plots. ‘Ramp-up’ refers to stimulation amplitude increasing from 0.5× to 1.0×, whereas ‘ramp-down’ refers to decreasing from 1.0× to 0.5×, during contralateral leg swing. **a**, Stacked bar graphs showing self-reported instances of falls and freezing episodes under each stimulation condition. The bars indicate the number of days that they had 0 (blue), 1 (green), 2–4 (orange) or 5+ (red) falls or freezing episodes. RU-aDBS reduced falls for patients 2 and 3, although it had little effect on freezing for patient 2 and increased the number of freezing episodes in patient 3. RD-aDBS had a similar effect, reducing falls while minimally affecting freezing episodes. Patient 4 did not report any falls or freezing under any condition. Group analysis using a cumulative link model indicated that ramp-up significantly increased the odds of reducing falls (OR = 4.35; *P* = 0.04; 95% CI = 1.07, 20.22). **b**, Stacked bar graphs illustrating subjective ratings of dyskinesia, patient-reported stiffness (as a surrogate for rigidity) and tremor. The bars show the number of days that each symptom was rated as ‘better’ (blue), ‘same’ (green) or ‘worse’ (red) compared to their baseline under clinically optimized cDBS before entering phase 4. The aDBS had little subjective impact for patients 2 and 3, with most days rated ‘same’ and only one instance of worsening patient-reported stiffness (patient 2). Patient 4 had a mixed response, including several ‘worse’ and two or more ‘better’ days. A cumulative link model revealed that patient-reported stiffness had a significantly higher likelihood of being rated ‘worse’ under both RU-aDBS (OR = 0.114; *P* = 0.017; 95% CI = 0.01, 0.59) and RD-aDBS (OR = 0.038; *P* = 0.0025; 95% CI = 0.00, 0.25). **c**, At-home gait metrics collected from a wearable triaxial ankle accelerometer (Rover) showing both RD-aDBS-improved and RU-aDBS-improved stride length and step symmetry relative to cDBS. Stride-length gains were greater with RD-aDBS (4.7%) than with RU-aDBS (0.9%), whereas step-asymmetry reductions were larger with RU-aDBS (2.37%) than with RD-aDBS (0.36%). Cadence and walking speed changes were minimal (0.51–1.41 steps min^−1^; 0.01–0.04 m s)^−1^), although patient 2 showed a significant (*P* = 0.045) increase in cadence compared with cDBS (+10.79 steps min^−1^). Data are presented as mean ± s.e.m for each metric. Comparison between stimulation therapies was performed using a two-sided Kruskal–Wallis test. Sample sizes are *n* = 23 for patients 2 and 3 and *n* = 19 for patient 4. **d**, Both RU-aDBS and RD-aDBS reducing right step-length variability (coefficient of variation (2–4%)) while increasing left step-length variability (1.4–1.5%). RD-aDBS decreased step-time variability bilaterally, whereas RU-aDBS produced opposing leg-specific effects. For **c** and **d**, patients are indicated by unique symbols (patient 2: square; patient 3: diamond; and patient 4: up-triangle).

Daily motor diaries captured falls, freezing episodes and self-rated changes in dyskinesia, tremor and perception of stiffness (as a surrogate for rigidity) relative to baseline (‘better’, ‘same’ and ‘worse’), with falls and freezing recorded in categorical bins (0, 1, 2–4 or 5+ events). At the group level, RU-aDBS was associated with a significant reduction in reported fall frequency compared to cDBS (odds ratio (OR) = 4.35, *P* = 0.047, 95% CI = 1.07, 20.22), indicating a higher likelihood of patients reporting lower fall-frequency categories (Fig. 5a). RD-aDBS had no significant effect. No group-level changes were observed for freezing episodes. In contrast, patients were less likely to report improved patient-reported stiffness on aDBS compared to cDBS (RU-aDBS: OR = 0.114, *P* = 0.018, 95% CI = 0.01, 0.60; RD-aDBS: OR = 0.038, *P* = 0.002, 95% CI = 0.00, 0.25) and no significant effects were observed for dyskinesia or tremor (Fig. 5b).

Individual-level responses revealed differences in patients. Patient 2 reported fewer falls under both aDBS strategies, with the largest reduction during RU-aDBS and a decrease in freezing episodes, although these remained frequent (2–4 per day). Patient 3, who rarely fell under cDBS, experienced further reductions in fall frequency during RU-aDBS relative to RD-aDBS, but freezing episodes increased under both strategies, most prominently with RU-aDBS. Patient 4 reported no falls or freezing episodes in any condition. Symptom ratings mirrored this variability: patients 2 and 3 reported stable daily scores across dyskinesia, tremor and patient-reported stiffness under all conditions, whereas patient 4 noted consistent improvement under cDBS but mixed responses to aDBS.

In addition to patient-reported motor diaries, blinded clinical assessments were administered in-clinic at the end of each testing phase (Extended Data Table 1). Comparing the three stimulation conditions, both RU-aDBS and RD-aDBS strategies generally maintained or improved global motor control relative to cDBS (MDS Unified Parkinson’s Disease Rating Scale part III (MDS-UPDRS-III)). Patients 2 and 3 exhibited improved (lower) motor scores under both aDBS conditions compared to cDBS, with RD-aDBS resulting in the lowest total motor score for patient 3 (26 versus 29). Conversely, patient 4 showed a slight worsening in motor scores under aDBS (+1–3 points). Postural stability scores (Mini-BESTest) improved while on RU-aDBS for patient 2 (+3 points versus cDBS), whereas patient 3 worsened under RU-aDBS. Patient 4 remained stable (21–22 points) across all conditions. Notably, Postural Instability and Gait Difficulty (PIGD) subscores remained largely stable across conditions for patients 2 and 4, but exhibited a slight improvement (reduction of 1–3 points) for patient 3.

Throughout the blinded aDBS testing period, all patient’s at-home gait metrics were captured and analyzed using customized analysis software from wearable triaxis accelerometers worn on their ankles (Rover, Sensoplex Inc.), which computed validated gait measurements^54^, including patient’s stride length (the distance traveled from one heel strike to the next), walking speed, cadence and step symmetry. On a group level, both aDBS conditions improved stride length and step symmetry compared to cDBS. For stride length, a larger effect was seen with RD-aDBS (4.7%) than with RU-aDBS (0.9%) (Fig. 5c). For step asymmetry, a RU-aDBS showed a greater reduction (2.37%) than RD-aDBS (0.36%). Cadence and walking speed were minimally affected by aDBS compared to cDBS, 0.51–1.41 steps min^−1^ and 0.01–0.04 m s^−1^, respectively. However, patient 2 exhibited a marked cadence increase, averaging +10.79 steps min^−1^ compared to their cDBS settings, but this change was not significant (*H*(2) = 5.89, *P* = 0.0525).

**Extended Data Fig. 2:**
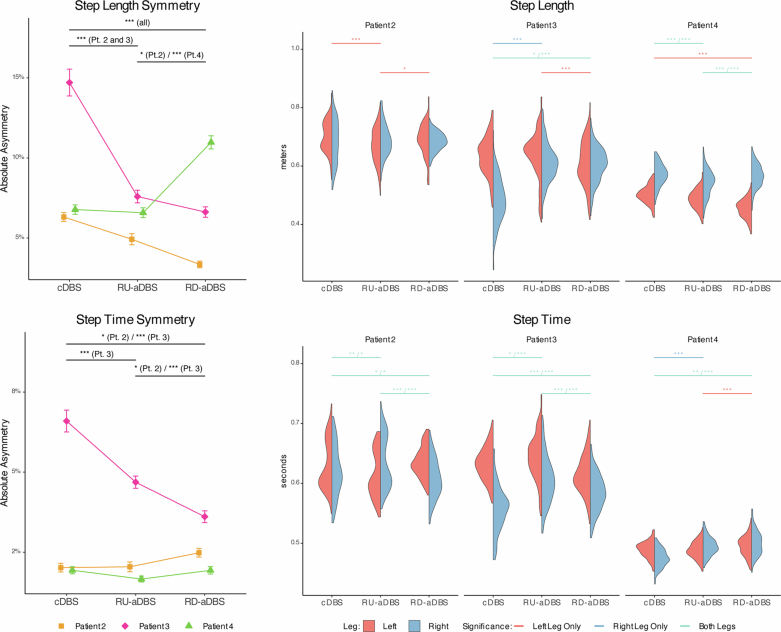
Long term in-clinic gait metrics. Double-blind testing in-clinic gait metrics. Data are presented as line plots (left column) or violin plots (right column). Line plots show mean±s.e.m. Violin plot color indicates left (red) or right (blue) leg. Comparison between clinically optimized DBS (cDBS), Ramp-up aDBS (RU-aDBS), and Ramp-down aDBS (RD-aDBS) were performed using a Kruskal-Wallis test for each patient individually with post-hoc analysis using Dunn’s test with Holm-Bonferroni p-value adjustments. Statistical testing for step length and time was stratified by leg. Outliers were defined at values beyond 1.5 time the interquartile range and were excluded from analysis. Sample sizes and statistical outcomes are detailed in Supplemental Tables 3 and 4. Significance level color indicates whether left (red), right (blue), or both (green) legs were significantly different. Significance levels are p<0.0005 (***), p<0.005 (**), and p<0.05 (*).

On the last day of each stimulation condition, gait metrics were calculated from in-clinic testing data. Both RU-aDBS and RD-aDBS consistently improved gait symmetry, with the largest improvements in step-length (2.3–2.9%) and smaller but consistent reductions in step-time asymmetry (0.7–0.8%) (Extended Data Fig. 2). Both aDBS strategies also reduced right step-length variability (2–4%) while increasing left step-length variability (1.4–1.5%) (Fig. 5d). Step-time variability changes were smaller and more variable, with RD-aDBS inducing bilateral reductions in step-time variability, whereas RU-aDBS elicited opposing effects between legs. Step-length and step-time values showed patient-specific patterns, including bilateral and unilateral changes, opposing leg effects and differences between step-length and step-time effects (Extended Data Fig. 2).

**Extended Data Fig. 3:**
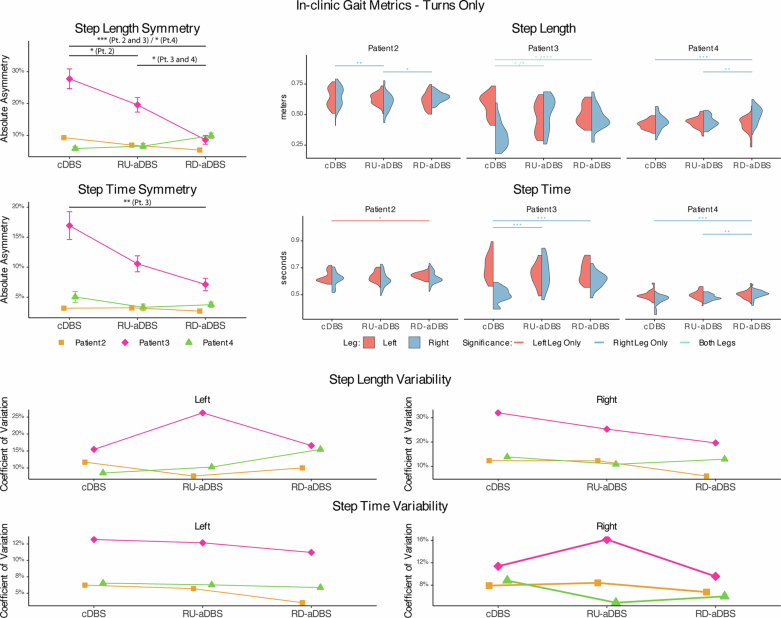
In-clinic gait metrics during turning in a randomized double-blinded comparison of cDBS compared to two aDBS settings. Comparison of continuous DBS (cDBS) with ramp-up (RU-aDBS) and ramp-down (RD-aDBS) adaptive stimulation was performed using a Kruskal-Wallis test for each patient followed by post-hoc analysis using Dunn’s test. P-values were adjusted for multiple comparisons using Holm-Bonferroni method. Outliers were defined at values beyond 1.5 time the interquartile range and were excluded from analysis. Sample sizes and statistical outcomes are detailed in Supplemental Tables 5 and 6. **Top left:** Plots showing absolute asymmetry for step length and step time. Colored lines represent individual patients (Patients 2, 3 and 4). Data are presented as mean ± s.e.m. **Top right:** Violin plots displaying the distribution of step length and step time for the left (red) and right (blue) legs for each patient across conditions. **Bottom:** Coefficient of variation for step length and step time. The analysis confirms that step length decreased and asymmetry increased, as biomechanically expected during turns, and was consistent across all conditions. This indicates that the system remained safe and effective despite the lack of turn-specific optimization.

As our study focused on identifying gait-phase biomarkers during straight-line walking, we found that there was variable behavior of the state classifier during turning. Although step length decreased and asymmetry increased during turns as biomechanically expected (Extended Data Table 2 and Extended Data Fig. 3), these changes were consistent across both aDBS and cDBS conditions, indicating that the system remained safe and effective despite the lack of turn-specific optimization.

## Discussion

Improving advanced gait disturbances with traditional DBS has remained a major clinical challenge. Here we developed and implemented a new gait-phase-synchronized aDBS therapy that rapidly modulates stimulation amplitude during contralateral leg swing on a subsecond timescale: a behavior-contingent aDBS paradigm that differs fundamentally from existing approaches. Personalized neural biomarkers, identified for each hemisphere, served as control signals for aDBS. These individualized biomarkers outperformed canonical frequency bands and demonstrated stability across testing sessions. Acute in-clinic testing revealed that aDBS reduced step-length and step-time variability and improved gait symmetry. Longer-term at-home testing confirmed not only aDBS’s tolerability and maintenance of motor symptom control, but also its efficacy for reducing falls and improving step length, cadence and symmetry compared to cDBS.

In this study, we introduced a gait-phase-synchronized aDBS paradigm that achieves subsecond, movement-locked modulation of stimulation amplitude, driven by individualized neural biomarkers derived from cortical–pallidal circuits. This represents a significant technical advance over prior aDBS approaches, which have largely operated on slower timescales (seconds to minutes)^46,55^ and targeted canonical β activity^47–49,56–58^ without consideration of the movement phase. Furthermore, this approach introduces a complementary control framework that operates on the timescale of behavior itself rather than as a direct alternative to existing state-based (for example, β driven) aDBS strategies. Given the highly dynamic nature of gait, the first feat was to identify reliable neural biomarkers of contralateral leg swing for each brain hemisphere. Instead of focusing on canonical frequency bands such as β, which is suppressed during voluntary and repetitive movement^59–61^ and therefore may lack specificity for gait, we adopted a data-driven approach to select patient-specific biomarkers. It is interesting that most personalized biomarkers fell within or spanned canonical frequency ranges, but showed superior performance for detecting leg swing compared to traditional bands (Fig. 2c).

The location of the best neural biomarker varied greatly between patients and also between the two brain hemispheres of patients, highlighting the need for multifocal network sensing that is necessary to implement our aDBS program. Our findings highlight two key advantages of incorporating cortical signals into aDBS control architectures. First, cortical recordings may provide superior decoding performance, because it provided the optimal control feature in five of the nine implanted hemispheres. Second, cortical electrodes offered substantially cleaner signals during stimulation, increasing robustness and reliability during closed-loop operation. By embedding patient-specific spectral features into a chronically implanted bidirectional neurostimulator, we achieved a second unique, technically challenging, accurate, real-time detection of contralateral leg swing and rapid ramping of stimulation amplitude within 100–500 ms, precisely timed to the kinematic demands of each step. Previous aDBS implementations typically operated over seconds, with slower ramping to avoid stimulation-induced feedback loops^62^. Despite these challenges, our biomarkers remained accurate and stable, demonstrating the feasibility of rapid aDBS without feedback instability. Our findings demonstrate that this level of control is both feasible and well tolerated in freely moving individuals with PD, and, to our knowledge, represent a novel implementation of fast aDBS synchronized to a movement event.

Beyond the engineering achievement, our results provide new insights into the circuit dynamics underlying gait control. We recorded robust, gait-phase-locked modulations across a broad range of frequencies—from θ and α to β and low γ—in both the pallidal and the motor cortical areas. These observations extend previous STN-based and cortex-based studies by implicating the pallidum as an active participant in phase-specific gait control, in synchrony with cortical rhythms. The heterogeneity in optimal biomarkers across patients suggests that gait control relies on individualized patterns of corticobasal ganglia coordination, shaped by disease progression, medication state and, perhaps, individual differences in gait strategies.

From a systems neuroscience perspective, the convergence of low-frequency rhythms linked to bilateral coordination and higher-frequency activity associated with motor execution supports a multi-band, distributed control model of locomotion. Recent evidence increasingly supports the view that gait is represented within distributed corticobasal ganglia circuits. Recordings from the STN and cortex in individuals with PD have demonstrated gait-phase-locked modulations in LFPs, particularly in low-frequency bands including θ, α and β^37,38,41,63,64^. These studies suggest that basal ganglia and cortical networks are dynamically engaged throughout the gait cycle, with distinct spectral modulations occurring during specific gait phases. Our findings align with and extend these observations. We recorded robust gait-phase-locked modulations in both the GPi and the motor cortex during natural overground walking, with modulations spanning a broad frequency range from θ through low γ (30–50 Hz). Although β-band activity has historically been emphasized due to its association with bradykinesia and rigidity, we observed that multiple frequency bands—including θ, α, β and low γ—were modulated during different phases of the gait cycle. In particular, lower-frequency rhythms such as θ and α, which have been associated with phasic muscle activation during locomotion^65,66^, may play a critical role in coordinating bilateral limb movements and maintaining rhythmic gait patterns. Given the need for precisely timed alternation of limb movements and balance adjustments during gait, lower-frequency synchronization across corticobasal ganglia circuits may contribute to the integration of bilateral motor outputs, whereas higher-frequency modulations may reflect more localized aspects of motor execution. Personalized biomarkers derived from these frequency ranges were highly effective in detecting contralateral leg swing and driving gait-phase-synchronized aDBS, further supporting their functional relevance. Future studies incorporating bilateral cortical and subcortical recordings will be important to delineate how different frequency bands cooperate across brain regions to support coordinated walking and how disruptions in these multi-band rhythms contribute to gait impairments in PD.

Clinically, gait-phase-synchronized aDBS produced improvements in step-length and step-time variability and bilateral symmetry—metrics that are closely tied to instability and fall risks^67–69^ and rarely improved with cDBS^70,71^. The mechanistic implication is that continuous stimulation, although beneficial for certain cardinal symptoms, may interfere with the physiological timing signals needed for dynamic balance and limb coordination. The ‘information lesion’ hypothesis posits that cDBS alleviates symptoms by disrupting pathological network communication^72–74^. Suppression of β activity after DBS supports this concept^75–83^. In the context of gait, this could mean suppression of critical sensorimotor cues that are phase locked to the walking cycle. Our approach preserves these windows for physiological processing by delivering stimulation only when pathological activity is most likely to impair performance—during specific phases of the gait cycle—while minimizing interference at other times. This temporally selective strategy likely explains why aDBS improved dynamic gait control more than cDBS.

The timing of stimulation may be key to modulating network dynamics. Current evidence suggests that pathological states can occur at different ends of network entropy. High entropy (that is, desynchronized activity) in basal ganglia activity may worsen parkinsonism^84–86^, whereas excessively low entropy is associated with hyperkinetic disorders^87^. This implies that effective neuromodulation requires pushing the network between these extremes. We hypothesized that gait-phase-synchronized aDBS introduces semi-random stimulation variability, modulating network entropy toward a functional equilibrium that supports effective gait control without destabilizing other motor functions.

A further advance in this work was the integration of long-term, at-home gait monitoring to assess real-world mobility. These data revealed benefits that were not consistently detectable during brief in-clinic testing, highlighting the considerable day-to-day variability in gait performance. Home-based assessment provides a more ecologically valid measure of function and captures the cumulative impact of therapy on fall risk and mobility. Crucially, although aDBS appeared to reduce fall frequency, we did not observe the same reduction in freezing episodes. This distinction highlights the value of continuous monitoring in dissecting specific symptom responses, suggesting that aDBS may primarily enhance gait stability rather than preventing freezing initiation. Our finding that aDBS may reduce falls in real-world conditions strengthens its translational relevance and points to the value of wearable sensor technology in both clinical trials and long-term patient management.

Our study has limitations. The small sample size reflects the invasive nature of device implantation and the stringent inclusion criteria for gait disturbances. In addition, although our platform allowed detection based on a single-frequency band, future implementations incorporating multiple bands may improve swing-phase detection accuracy^55^. The classifier successfully rejected nongait movements, including voluntary limb movements in patients 2–5 and dyskinetic episodes in patient 1. However, future work is needed to validate classifier performance in a larger cohort with severe, distinct dyskinetic patterns. Furthermore, our assessment of at-home safety was limited to a self-reported motor diary. Objective quantification of near falls was not possible because of the lack of validated automated detection standards for these events. Finally, clinical response was heterogeneous. Patient 1, who exhibited severe dyskinesia, showed no improvements in gait metrics, possibly due to the chronic instability of stimulation settings and limited acute testing duration. These findings highlight the need for longer adaptation periods in future studies.

Our results demonstrate that subsecond, gait-phase-synchronized aDBS—driven by individualized cortical–pallidal biomarkers—can restore dynamic gait control in PD while preserving broader motor function. By targeting stimulation to the precise temporal windows when pathological activity is most disruptive, this approach overcomes the key limitations of cDBS, extends adaptive neuromodulation beyond conventional β-state-based paradigms and reveals mechanistic insights into the circuit-level control of locomotion. The integration of home-based gait monitoring further establishes its real-world relevance. These findings position movement-state-driven aDBS as a viable and potentially transformative therapy for gait disorders, paving the way toward a new class of precision-timed neuromodulation strategies that aim not just to suppress symptoms, but also to re-establish the physiological dynamics of human movement.

## Methods

### Clinical trial overview

This study was completed within the ‘Adaptive deep brain stimulation to improve motor and gait functions in Parkinson’s disease’ clinical trial (CclinicalTrials.gov: NCT04675398; 17 December 2020). The primary outcome of this trial is to deliver personalized neurostimulation based on individual physiological biomarkers to enhance locomotor skills in patients with PD. All data presented here are part of the ongoing exploratory clinical trial and do not contribute toward any conclusions about the primary outcome of the trial. The neural device used in this study (Medtronic Summit RC + S) was approved by the US Food and Drug Administration as an investigation device exemption, which was followed by study protocol approval by the University of California, San Francisco (UCSF) Institutional Review Board (protocol no. 20-32847, 11 June 2021). Five patients gave their informed consent to participate in this trial after multiple conversations with study investigators in which the details of study enrollment, including risks related to the study device, were explained to them.

### Patients

We recruited four patients (three men, one woman, aged 68, 64, 68 and 68 years) with PD who were undergoing DBS between June 2021 and October 2022 and one patient (man, aged 53 years) joined our study while he was enrolled in another clinical trial (NCT03582891). The reported sex of the patients in our study is based on the sex assigned at birth. As we only had one patient who identified as female, no sex-based and gender-based analyses have been performed. We acknowledge the importance of including both sexes in future research. Inclusion criteria included age 18–75 years, idiopathic PD (≥3 years), MDS-UPDRS-III OFF scores 20–80 with ≥30% medication response, Montreal Cognitive Assessment ≥21 and gait impairments (slowed gait, shuffling steps, postural instability or freezing of gait off medication). Additional criteria are given in Table 1. Exclusion criteria were untreated depression (Beck Depression Inventory II >20), active suicidal ideation, history of cranial surgery or other implanted stimulation systems. All patients were recruited from outpatient neurology or neurological surgery clinics at UCSF.

### Neural implants and lead reconstruction

The investigational neural device used in this study (Medtronic Summit RC + S, B35300R) is a rechargeable neural interface, capable of simultaneously delivering stimulation to the DBS target and recording neural LFPs. Up to four bipolar electrode pairs can be recorded from. In addition, it is capable of a fully embedded adaptive algorithm.

Four patients (1, 2, 3 and 5) underwent bilateral implantation and one patient (4) underwent left hemisphere unilateral implantation of a cylindrical quadripolar DBS lead into the GPi (Medtronic, model 3387) and a quadripolar cortical paddle over the cortex (Medtronic, model 0913025). The cortical paddles were positioned such that the electrodes spanned over the M1 and PM cortices in patients 1–4, whereas in patient 5 they were positioned over M1 and S1. A DBS and cortical paddle leads from the same hemisphere were connected to each Summit RC + S neural stimulator.

Precise electrode localization was performed using established image analysis pipelines for depth and cortical electrodes^29^. Post-implantation computed tomography was coregistered to preoperative, T1-weighted, 3T magnetic resonance imaging via rigid affine transformation. Accuracy was verified visually and corrected for brain shift where necessary. Then, electrode artifacts were identified on the computed tomography images and fitted to known electrode geometry. Cortical electrodes were additionally projected to the magnetic resonance imaging-rendered pial surface. Last, electrode locations were normalized into Montreal Neurological Institute space^88^ and visualized on either the FreeSurfer average cortical surface^89^ or a PD-specific standard subcortical atlas^90^.

### Experimental paradigm

Patients 1–4 were brought into our lab, located at UCSF, twice before aDBS testing. The first research visit occurred between 26 d and 43 d post-implantation before turning on DBS, and the second visit occurred once a UCSF neurologist had determined their clinically optimized DBS parameters (140–520 d). Patient 5, who joined our study after being clinically optimized, was brought into the lab once before aDBS testing. Each visit, the patients were instructed to walk overground at their self-selected speed for 250 steps. This was repeated in the ON-medication state and in a LOW-medication state. A LOW-medication state was defined as 15 min before their next medication dose. Data from these visits were used to generate patient-specific candidate biomarkers to detect the contralateral leg swing.

The biomarkers served as the control signal for our embedded aDBS algorithm, which aimed to change the stimulation amplitude from their clinically optimized stimulation amplitude during contralateral leg swing to half the value during all other gait phases (Fig. 1a). The stimulation frequency, pulse width and electrode contacts did not change from their clinically optimized values to minimize the side-effect potential. Each biomarker was evaluated for accuracy by turning on aDBS for 15–20 min, asking the patient to perform the walking task above and comparing the output of the DBS system with the timings of the contralateral leg swing. Biomarkers with the highest accuracies were tested again to optimize parameters such as the threshold and how fast the stimulation amplitude would increase or decrease. More details on how these biomarkers were determined, evaluated and optimized can be found below.

Optimized aDBS settings for patients 2, 3 and 4 were tested in a double-blind experiment by randomizing three conditions: cDBS; aDBS ramping from 0.5× to 1.0× the clinically optimized amplitude value during contralateral leg swing (RU-aDBS); and aDBS ramping from 1.0× to 0.5× the clinically optimized amplitude value during contralateral leg swing (RD-aDBS). Complete block randomization was generated using a computer before the initiation of double-blind testing by J.H.M., who was the only research personnel aware of each patient’s assigned condition during this period. Each condition was maintained in every patient for 8–10 d. At the start of each condition, J.H.M. adjusted the aDBS parameters to ensure that all other researchers remained blinded. During testing, patients wore ankle accelerometer devices (Rover, Sensoplex Inc.), each containing a sensor comprising a triaxial gyroscope, accelerometer and magnetometer that recorded kinematic data locally. This data were collected passively after bilateral ankle placement and uploaded to the manufacturer’s cloud software for data processing and analysis of gait metrics. Patients also completed post-test motor diaries in the evening.

### Data acquisition

Gait kinematics were collected from the patients using two wireless systems: the Delsys Trigno or Centro (Delsys Inc.) and Xsens MVN Analyze (Movella). The Delsys system included: eight inertial measurement unit + electromyography sensors placed bilaterally on top of their neural implant, hands, tibialis anterior and soleus; two goniometers (SG110/A) placed next to the lateral malleolus; and eight force-sensitive resistors (FSRs; DC:F01) placed bilaterally on the toe, first metatarsal, fifth metatarsal and heel^37^. The Xsens system comprised 15 inertial measurement unit sensors which were placed in accordance with Xsens instructions for wireless motion tracking.

Neural LFPs were recorded from one subcortical and two cortical bipolar electrode pairs. GPi potentials were recorded from contacts adjacent to the stimulating cathode, allowing for common mode rejection of the stimulation artifact when stimulation was being delivered. Cortical recordings were recorded from adjacent pairs with the anterior pair overlaying the PM cortex. LFPs were sampled at 500 Hz (0.85 Hz high-pass) and low-pass filtered at 100 Hz (subcortical) or 450 and 1,700 Hz (cortical). Triaxial accelerometry data were also collected from the Summit RC + S system and sampled at 64 Hz. All data from the Summit RC + S system were extracted and processed using the OpenMind consortium open-source code (https://github.com/openmind-consortium/Analysis-rcs-data)^55,91^.

### Data analysis

Neural LFP data collected before aDBS were inspected for stimulation, electrocardiogram (ECG) and nonphysiological polyphasic artifacts. Stimulation artifacts were filtered using a fourth-order Butterworth filter at the patient’s clinically optimized stimulation frequency and at half the value to remove harmonics. ECG artifacts were removed using a template subtraction method by providing a manually selected ECG artifact^92^. In rare cases, a nonphysiological polyphasic artifact would be present and was removed using the same template subtraction method to remove ECG artifacts.

Gait events were extracted from the toe and heel FSRs using a customized MATLAB script. Heel strikes were defined as the time when the heel FSR reading was >5% in the positive direction. Toe-offs were defined as the time when the toe FSR reading was <5% in the negative direction. In some instances, gait events were estimated using signals from the other intact sensors when toe or heel FSRs were damaged during an overground walking bout.

Filtered neural LFPs, gait events and gait kinematic data were synchronized by aligning the peaks in acceleration captured by the Summit RC + S device(s), Delsys sensors over the Summit RC + S and Xsens acceleration around the shoulders^37^. Wavelet spectral analysis (MATLAB cwt function) was applied to the LFP signals and the power spectra for each gait cycle were extracted and normalized to the average power during movement. Separately, the output of the Summit RC + S FFT was simulated using the raw LFP signals with the Python rcssim package^93^ (https://github.com/Weill-Neurohub-OPTiMaL/rcs-simulation). Spectral power during the left and right leg-swing phase was calculated within the θ (4–7 Hz), α (7–12 Hz), β (13–30 Hz) and low γ (30–50 Hz), to see whether any bands exhibited a power difference.

In-clinic gait metrics were derived from recordings obtained with the Delsys and Xsens systems and were used as our primary metrics to evaluate changes in gait from cDBS to aDBS. Temporal metrics included step time, swing time and double support time, extracted from gait events detected by FSRs of the Delsys system. Step length was the only spatial metric and was calculated from foot position data recorded by an Xsens sensor placed on top of each patient’s shoe. Step time was defined as the interval from heel strike of one foot to heel strike of the contralateral foot. Step length was defined as the distance from the heel of one foot to the heel of the contralateral foot at heel strike.

Step length and time symmetry were calculated as:

1 $${\rm{Symmetry}}=\,\frac{{\rm{Left}}\,{\rm{leg}}-{\rm{Right}}\,{\rm{leg}}}{{\rm{Left}}\,{\rm{leg}}+{\rm{Right}}\,{\rm{leg}}}$$

where 0 represents perfect symmetry, a positive value a longer left step and a negative value a longer right step.

At-home gait data were recorded via Rover wearable modules, uploaded to a secure cloud and processed into reports summarizing stride duration, length and swing periods. These reports were used to calculate average gait metrics for all patients during the long-term aDBS phase.

### Statistical analysis

Differences in simulated Summit RC + S spectral power during the left and right swing phases were tested for each patient, frequency band, hemisphere and recording location separately. Normality was first tested for each group using the Shapiro–Wilk test, with all tests achieving a *P* < 0.05, indicating that all extracted spectral power data were not normally distributed. Significance testing for each group was performed using the two-sided Wilcoxon’s rank-sum test.

Biomarker spectral power, computed from the Summit RC + S, for each patient was compared at three different timepoints: before and after optimization of clinical DBS parameters and during aDBS testing. A Shapiro–Wilk test determined that the spectral power was not normally distributed. Thus, a Kruskal–Wallis test was performed across the three timepoints. To determine which timepoints are significantly different from each other, a post-hoc analysis was performed using two-sided Dunn’s test with a Holm–Bonferroni *P*-value adjustment for multiple comparisons.

The effect sizes were calculated across the three timepoints using unpaired Cohen’s *d* for each comparison, separated by hemisphere, to quantify the magnitude of the distribution shifts. After excluding outliers falling outside 1.5× the IQR, differences of power between pairs of timepoints were classified as negligible, small, moderate and large^52^. The 95% CIs for the effect size were estimated by bootstrapping >1,000 iterations.

Temporal and spatial gait metrics and symmetry changes from the cDBS to the aDBS setting were tested for each patient. Values were marked as outliers if they were not contained within 1.5× the IQR and removed. Statistical testing followed the same scheme, where normality was tested first, indicating that these metrics were not normally distributed, and then a two-sided Wilcoxon’s rank-sum test (during phase 2) or a Kruskal–Wallis test (during phase 3) was performed to compare all DBS settings. Turns were excluded from all analysis, because this study focused on straight-line overground walking.

Patients completed end-of-day motor diaries during phase 4 to rate the severity of three cardinal PD symptoms—dyskinesia, perception of stiffness (as a surrogate for rigidity) and tremor—relative to their baseline experience under clinically optimized cDBS and recall the number of falls and freezing episodes that they experienced during the day. Ratings for the cardinal symptoms were recorded on a three-point ordinal scale: ‘worse’, ‘same’ or ‘better’. For the number of falls and number of freezing episodes, patients selected one of four categories: 0, 1, 2–4 or 5+. To evaluate whether stimulation conditions influenced symptom perception, the number of falls or the number of freezing episodes, a cumulative link model was used to assess the probability of reporting a change from cDBS across conditions. The category order was defined as follows: ‘worse’, ‘same’ or ‘better’ for patient-reported stiffness, bradykinesia and tremor; and 5+, 2–4, 1 and 0 for the number of falls and freezing episodes.

### Adaptive DBS

The adaptive algorithm was designed to increase or decrease the stimulation amplitude while keeping the stimulation frequency, pulse width and stimulating electrode contact the same. The stimulation amplitude ranged from 0.5× to 1.0× the patient’s clinically optimized therapeutic value. This range was selected to maintain a continuous therapeutic baseline for other cardinal motor symptoms during low-amplitude phases, because prior research has indicated limited efficacy with a broader 0–1.0× range^94^. Changes in stimulation amplitude were targeted to occur only during contralateral leg swing (Fig. 1a).

Embedding the algorithm into the Summit RC + S system involved setting several values: the biomarker frequency band(s), the rate of change for the stimulation amplitude (both increases and decreases), the onboard FFT parameters, the state parameters and the threshold for changing states^55,62^. Here ‘state’ refers to a set of stimulation parameters that the Summit RC + S system will adjust to based on whether the short-time Fourier transform power of the biomarker is above or below the threshold. Our algorithm used a single-frequency band, which required us to set two states and a single threshold value. After setting these values, the biomarker was evaluated for accuracy and, if it showed the highest accuracy, the threshold and rate of change were further optimized.

#### Gait phase biomarker search

A data-driven approach was used to determine which frequency band to test as the biomarker for the adaptive algorithm. Candidate biomarkers were identified by simulating arbitrary length frequency bands between 2.5 Hz and 50 Hz using the rcssim package^93^, evaluating 15 thresholds (15–85% quantiles) for gait-phase detection accuracy. The 20 biomarkers with the highest accuracies were then selected to be tested with the patients.

#### Biomarker evaluation

Candidate biomarkers were embedded into the patients’ left and right Summit RC + S systems and tested one at a time. Patients were sat in a chair with armrests for 1 min after aDBS was activated, during which they informed the research team of any side effects. If a side effect did not subside within the next minute, the biomarker trial was stopped, the patient was returned to their clinically optimized setting and the next biomarker was tested. Across all patients, three side effects were reported: patient 1 experienced dyskinesia primarily affecting the right side of their body, patient 3 experienced visual abnormalities and patient 4 experienced muscle pulling around the right jaw. Once the patients were returned to their clinical DBS settings, the side effects ceased.

Biomarker settings that did not cause side effects continued the testing with 1 min of standing arm swing and overground walking at a self-selected speed for 250 steps. During the overground walking portion, a licensed physical therapist (J.E.B.) guarded the patient to prevent any falls. Overground walking data were then processed offline and analyzed for accurate changes in stimulation amplitude during the contralateral swing phase. Accuracy was calculated by summing the total time that the patient spent in contralateral leg swing (detected by the Summit RC + S system as a true positive) and the total time spent in other gait phases (when the Summit RC + S system correctly did not detect swing phase as a true negative). This sum was then divided by the total length of the trial:

2 $${\rm{Accuracy}}=\frac{{\rm{Total}}\,{\rm{time}}\,{\rm{in}}\,{\rm{true}}\,{\rm{positive}}+{\rm{Total}}\,{\rm{time}}\,{\rm{in}}\,{\rm{true}}\,{\rm{negative}}}{{\rm{Total}}\,{\rm{trial}}\,{\rm{time}}}.$$

#### Biomarker optimization

The biomarkers that achieved the highest accuracy for the left and right Summit RC + S systems were tested in the next adaptive testing session to optimize the threshold, the rate of change of the stimulation amplitude and the state parameters. Thresholds were increased or decreased based on visual inspection of the Summit RC + S-reported stimulation amplitude during overground walking. Across all patients, threshold adjustments were within ±10% of the original threshold predicted by the data-driven simulation. The rate of change for the stimulation amplitude was initially set to 1.9–2.7 mA per 400 ms, depending on the patient’s clinically optimized value and was reduced if side effects were observed. Last, the two states were switched if the amplitude decreased during the contralateral leg swing. The optimization process required a mean of 6.4 visits per patient (range: 1–10), with each visit lasting approximately 5 h.

#### Double-blind testing

The optimized adaptive setting, referred to as RU-aDBS, was evaluated in a double-blind study against each patient’s clinically optimized setting and an additional adaptive setting, RD-aDBS. The ramp-down protocol reversed the ramp-up stimulation pattern by decreasing amplitude from 1.0× to 0.5× during contralateral leg swing. Each setting was tested over 8–10 d. At the end of each day, patients completed a motor diary rating dyskinesia, perception of stiffness (as a surrogate for rigidity) and tremor relative to their baseline under cDBS. They also recorded the number of falls and freezing episodes experienced. Together, these motor diary ratings were used to evaluate secondary effects of aDBS compared to cDBS. Last, on the final day of each setting, patients returned to the clinic for an in-person research assessment.

### Reporting summary

Further information on research design is available in the Nature Portfolio Reporting Summary linked to this article.

## Online content

Any methods, additional references, Nature Portfolio reporting summaries, source data, extended data, supplementary information, acknowledgements, peer review information; details of author contributions and competing interests; and statements of data and code availability are available at 10.1038/s41591-026-04434-2.

## Supplementary information

Supplementary Information Supplementary Tables 1–6.

Reporting Summary

Peer Review File

Supplementary Video 1 Example of gait phase aDBS running in patient 2. Xsens motion capture video is shown on the left. Summit RC + S amplitude, gait phase biomarker power and detector state is shown on the left. Additionally, left (blue) and right (purple) leg swing phase are shown as colored boxes.

Supplementary Video 2 Patient 3 performs left and right finger tapping while seated on aDBS, demonstrating stable performance of the embedded classifier. For this patient, state 1 is defined as their non-walking state.

Supplementary Video 3 Patient 1 demonstrates foot dyskinesia while seated during aDBS, with the embedded classifier remaining stable. For this patient, state 1 is defined as their non-walking state.

Supplementary Video 4 Video of patient 2 on cDBS and RU-aDBS. The camera was positioned differently from the Xsens recording system. As a result, the animation and camera footage show the subject walking in opposite directions.

## Data Availability

Due to the inclusion of identifiable clinical and neurophysiological data from human participants, the data were subject to controlled access to protect participant privacy and comply with institutional and regulatory requirements. Requests for access should be directed to the corresponding author and will be reviewed within a reasonable timeframe (typically within 2–4 weeks), contingent on institutional approvals and data use agreements that restrict re-identification and redistribution. Requests will be reviewed based on scientific merit, ethical review and available resources. Following approved requests, de-identified individual patient data will be made available for reuse. The data used to generate the figures in this paper are available at https://dabi.loni.usc.edu/projects/F3RE38A785JW.

## Extended data

**Extended Data Table 1:** Double-blind aDBS Clinical Scores

	cDBS MDS-UPDRS/PIGD	cDBS Mini-BESTest	RU-aDBS MDS-UPDRS/PIGD	RU-aDBS Mini-BESTest	RD-aDBS MDS-UPDRS/PIGD	RD-aDBS Mini-BESTest
Patient 2	26/3	18	21/3	21	20/3	17
Patient 3	29/6	21	27/5	18	26/3	21
Patient 4	11/1	22	12/1	21	14/2	22

cDBS = continuous DBS, RU-aDBS = Ramp-up aDBS, RD-aDBS = Ramp-down aDBS, PIGD = Total of UPDRS III items: 3.9–3.11 and 3.13

**Extended Data Table 2:** Double-blinded cDBS vs aDBS Gait Metrics During Turning

		Step Length (m)			Step Time (s)		
		µ ± SD	CV	Absolute Asymmetry	µ ± SD	CV	Absolute Asymmetry
**Patient 2**							
cDBS	*Left*	0.64±0.07	0.12	9.3%	0.63±0.04	0.06	3.2%
	*Right*	0.65±0.08	0.12		0.62±0.05	0.08	
RU-aDBS	*Left*	0.63±0.05	0.07	6.9%	0.64±0.04	0.06	3.3%
	*Right*	0.60±0.07	0.12		0.62±0.05	0.08	
RD-aDBS	*Left*	0.62±0.06	0.10	5.4%	0.64±0.02	0.04	2.7%
	*Right*	0.64±0.04	0.06		0.63±0.04	0.07	
**Patient 3**							
cDBS	*Left*	0.58±0.09	0.15	27.8%	0.70±0.09	0.13	17.0%
	*Right*	0.33±0.11	0.32		0.50±0.06	0.11	
RU-aDBS	*Left*	0.47±0.12	0.26	19.6%	0.65±0.08	0.13	10.6%
	*Right*	0.52±0.13	0.25		0.66±0.11	0.16	
RD-aDBS	*Left*	0.49±0.08	0.17	8.6%	0.67±0.07	0.11	7.1%
	*Right*	0.47±0.09	0.19		0.61±0.06	0.10	
**Patient 4**							
cDBS	*Left*	0.42±0.04	0.09	5.8%	0.50±0.03	0.07	5.0%
	*Right*	0.44±0.06	0.14		0.46±0.04	0.09	
RU-aDBS	*Left*	0.44±0.04	0.10	6.6%	0.50±0.03	0.06	3.3%
	*Right*	0.44±0.05	0.11		0.48±0.02	0.05	
RD-aDBS	*Left*	0.42±0.06	0.15	9.9%	0.50±0.03	0.06	1.2%
	*Right*	0.50±0.06	0.13		0.50±0.03	0.06	

cDBS = Continuous Deep Brain Stimulation

RU-aDBS = Ramp-up aDBS

RD-aDBS = Ramp-down aDBS

CV = Coefficient of Variation

SD = Standard Deviation.

## Code availability

Except for the biomarker identification code, all MATLAB, Python and R analysis code used to analyze and generate the main findings of this study will be made available at https://github.com/UCSF-wang-lab/gp-FaDBS within 1 month of publication. The biomarker identification code will be made available after subsequent manuscripts that use this code have been published. Code written to interface with and process raw data from the Medtronic Summit RC + S is available in the OpenMind public GitHub repository (https://openmind-consortium.github.io).
