# Supplementary material for: Adaptive deep brain stimulation for dynamic gait control in Parkinson’s disease: a randomized feasibility trial
Source: Nat Med. 2026 Jun 15;32(8):2803–14. doi: 10.1038/s41591-026-04434-2 (PMC13473014; doi:10.1038/s41591-026-04434-2)
Supplement: Supplementary file 1 [file 41591_2026_4434_MOESM1_ESM.pdf]

nature medicine

Supplementary information https://doi.org/10.1038/s41591-026-04434-2

Adaptive deep brain stimulation for dynamic gait control in Parkinson's disease: a randomized feasibility trial

In the format provided by the authors and unedited

Supplementary Table 1: Statistic for Patient-specific aDBS biomarker distributions pre-optimized DBS, post-optimized DBS, and aDBS testing (**Fig. 3c**). List of Cohen's D values and 95% confidence intervals in brackets.

	**Left**			**Right**		
N	*Pre*	*Post*	*aDBS*	*Pre*	*Post*	*aDBS*
Patient 1	-	3108	1552	-	1618	948
Patient 2	1875	732	201	2324	2280	650
Patient 3	1449	1735	417	439	684	132
Patient 4	925	971	827	-	-	-
Patient 5	-	2325	356	-	2472	890
	Pre-Post	*Pre-aDBS*	*Post-aDBS*	*Pre-Post*	*Pre-aDBS*	*Post-aDBS*
Patient 1	-	-	*d* = 0.039 [-0.02,0.10]	-	-	*d* = -1.38 [-1.50,-1.28]
Patient 2	*d* = -1.78 [-1.90,-1.67]	*d* = -0.92 [-1.08,-0.76]	*d* = 0.62 [0.45,0.81]	*d* = 0.88 [0.03,0.15]	*d* = -0.59 [-0.68,-0.51]	*d* = -0.68 [-0.76,-0.6]
Patient 3	*d* = 0.21 [0.14,0.28]	*d* = -0.428 [-0.53,-0.32]	*d* = -0.65 [-0.76,-0.55]	*d* = 0.25 [0.13,0.36]	*d* = -0.38 [-0.57,-0.18]	*d* = -0.64 [-0.83,-0.45]
Patient 4	*d* = 0.72 [0.64,0.82]	*d* = -0.40 [-0.49,-0.30]	*d* = -1.14 [-1.24,-1.05]	-	-	-
Patient 5	-	-	*d* = -0.67 [-0.79,-0.56]	-	-	*d* = -1.01 [-1.09,-0.93]

Supplementary Table 2: Statistics for gait metric comparison between Patient's clinically optimized DBS and aDBS (**Fig. 4**). List of sample sizes, Wilcoxon test statistics, and p-values.

Group Statistics

	N (cDBS/aDBS)	*W* (*p-value*)
**Step Length Symmetry**	423/383	109982 ($p<0.0001$)
**Step Time Symmetry**	476/390	100426 ($p<0.05$)
**Step Length Variability**		
Left	5	19 ($p=0.22$)
Right	5	19 ($p=0.22$)
**Step Time Variability**		
Left	5	22 ($p=0.056$)
Right	5	18 ($p=0.31$)

Individual Statistics

	Patient 1	Patient 2	Patient 3	Patient 4	Patient 5
**Step Length Symmetry**					
N (cDBS/aDBS)	48/176	162/85	88/40	59/40	66/42
W (*p-value*)	*7401* ($p=0.33$)	*958* ($p<0.0001$)	*2067* ($p<0.0001$)	206 ($p<0.0001$)	1008 ($p<0.0001$)
**Step Time Symmetry**					
N (cDBS/aDBS)	189/91	102/40	70/40	53/42	62/177
W (*p-value*)	6111 ($p<0.0001$)	3966 ($p<0.0001$)	1643 ($p=0.13$)	0 ($p<0.0001$)	7140 ($p<0.001$)
**Step Length**					
Left					
N (cDBS/aDBS)	181/91	102/40	64/41	66/62	72/177
W (*p-value*)	15123 ($p<0.0001$)	2612 ($p<0.05$)	1590 ($p=0.07$)	2495 ($p<0.05$)	1299 ($p<0.0001$)
Right					
N (cDBS/aDBS)	186/85	102/40	61/40	66/60	68/177
W (*p-value*)	10406 ($p<0.0001$)	3568 ($p<0.0001$)	321 ($p<0.0001$)	3757 ($p<0.0001$)	9036 ($p<0.0001$)
**Step Time**					
Left					
N (cDBS/aDBS)	189/124	133/40	72/50	68/62	90/177
W (*p-value*)	5890 ($p<0.0001$)	5313 ($p<0.0001$)	3088 ($p<0.0001$)	28 ($p<0.0001$)	15430 ($p<0.0001$)
Right					
N (cDBS/aDBS)	239/94	136/48	93/40	96/81	86/178
W (*p-value*)	9530 ($p<0.05$)	3506 ($p=0.45$)	3117 ($p<0.0001$)	7120 ($p<0.0001$)	11422 ($p<0.0001$)

Supplementary Table 3: Kruskal-Wallis test statistics for in-clinic gait metrics comparison during Phase 3 of the study (**Extended Data Fig. 2**, double-blind aDBS testing). Comparison was performed across clinically optimized DBS (cDBS), Ramp-Up aDBS (RU-aDBS), and Ramp-Down aDBS (RD-aDBS)

**Kruskal-Wallis Sample Sizes and Statistics**		
**Patient 2 (N = 467)**		
	Left	*Right*
Step Length	$H(2) = 19.8$, $p<0.0001$	$H(2) = 0.4$, $p=0.8$
Step Time	$H(2) = 29.6$, $p<0.0001$	$H(2) = 24.7$, $p<0.0001$
	Combined	
Step Length Symmetry	$H(2) = 60.3$, $p<0.0001$	
Step Time Symmetry	$H(2) = 12.0$, $p<0.05$	
**Patient 3 (N = 605)**		
	Left	*Right*
Step Length	$H(2) = 19.7$, $p<0.0001$	$H(2) = 178.5$, $p<0.0001$
Step Time	$H(2) = 79.8$, $p<0.0001$	$H(2) = 107.8$, $p<0.0001$
	Combined	
Step Length Symmetry	$H(2) = 79.2$, $p<0.0001$	
Step Time Symmetry	$H(2) = 59.0$, $p<0.0001$	
**Patient 4 (N = 565)**		
	Left	*Right*
Step Length	$H(2) = 147.4$, $p<0.0001$	$H(2) = 43.8$, $p<0.0001$
Step Time	$H(2) = 19.2$, $p<0.0001$	$H(2) = 160.4$, $p<0.0001$
	Combined	
Step Length Symmetry	$H(2) = 77.1$, $p<0.0001$	
Step Time Symmetry	$H(2) = 3.07$, $p = 0.21$	

Supplementary Table 4: Post-hoc Dunn's test statistics with Holm-Bonferroni p-value adjustment for in-clinic gait metrics comparison during Phase 3 of the study (**Extended Data Fig. 2**, double-blind aDBS testing). Comparison were performed across clinically optimized DBS (cDBS), Ramp-Up aDBS (RU-aDBS), and Ramp-Down aDBS (RD-aDBS).

**Dunn's Test Sample Sizes and Statistics**			
	cDBS	RU-aDBS	RD-aDBS
Patient 2	174	139	154
Patient 3	118	249	238
Patient 4	195	204	165

**Comparisons**						
	**cDBS – RU-aDBS**		**cDBS – RD-aDBS**		**RU-aDBS – RD-aDBS**	
Patient 2	Left	Right	Left	Right	Left	Right
Step Length	$z = -4.41$ ($p<0.0001$)	-	$z = -1.50$ ($p=0.13$)	-	$z = 2.87$ ($p<0.05$)	-
Step Time	$z = -3.55$ ($p<0.001$)	$z = 2.48$ ($p<0.05$)	$z = 2.04$ ($p<0.05$)	$z = -2.70$ ($p<0.05$)	$z = 5.38$ ($p<0.0001$)	$z = -4.96$ ($p<0.0001$)
Step Length Symmetry	$z = -4.18$ ($p<0.0001$)		$z = -7.73$ ($p<0.0001$)		$z = -3.24$ ($p<0.05$)	
Step Time Symmetry	$z = 0.21$ ($p = 0.83$)		$z = 3.18$ ($p<0.05$)		$z = 2.80$ ($p<0.05$)	
Patient 3	Left	Right	Left	Right	Left	Right
Step Length	$z = 0.80$ ($p = 0.42$)	$z = 12.4$ ($p<0.0001$)	$z = -2.69$ ($p<0.05$)	$z = 12.0$ ($p<0.0001$)	$z = -4.32$ ($p<0.0001$)	$z = -0.41$ ($p=0.70$)
Step Time	$z = 2.38$ ($p<0.05$)	$z = 10.3$ ($p<0.0001$)	$z = -4.76$ ($p<0.0001$)	$z = 5.78$ ($p<0.0001$)	$z = -8.85$ ($p<0.0001$)	$z = -5.49$ ($p<0.0001$)
Step Length Symmetry	$z = -7.61$ ($p<0.0001$)		$z = -8.53$ ($p<0.0001$)		$z = -1.21$ ($p = 0.23$)	
Step Time Symmetry	$z = -4.43$ ($p<0.0001$)		$z = -7.62$ ($p<0.0001$)		$z = -4.01$ ($p<0.0001$)	
Patient 4	Left	Right	Left	Right	Left	Right
Step Length	$z = -4.46$ ($p<0.0001$)	$z = -6.20$ ($p<0.0001$)	$z = -12.0$ ($p<0.0001$)	$z = -0.96$ ($p=0.34$)	$z = -7.89$ ($p<0.0001$)	$z = 4.96$ ($p<0.0001$)
Step Time	$z = -0.57$ ($p = 0.57$)	$z = 10.2$ ($p<0.0001$)	$z = 3.52$ ($p<0.001$)	$z = 11.5$ ($p<0.0001$)	$z = 4.11$ ($p<0.001$)	$z = 1.91$ ($p=0.57$)
Step Length Symmetry	$z = -0.53$ ($p = 0.59$)		$z = 7.41$ ($p<0.0001$)		$z = 8.00$ ($p<0.0001$)	
Step Time Symmetry	-		-		-	

Supplementary Table 5: Kruskal-Wallis test statistics for in-clinic gait metrics during turns comparison during Phase 3 of the study (**Extended Data Fig. 3**, double-blind aDBS testing). Comparison was performed across clinically optimized DBS (cDBS), Ramp-Up aDBS (RU-aDBS), and Ramp-Down aDBS (RD-aDBS)

**Kruskal-Wallis Sample Sizes and Statistics**		
**Patient 2 (N = 217)**		
	Left	*Right*
Step Length	$H(2) = 4.01$, $p=0.14$	$H(2) = 17.1$, $p<0.001$
Step Time	$H(2) = 7.72$, $p<0.05$	$H(2) = 3.02$, $p=0.22$
	Combined	
Step Length Symmetry	$H(2) = 24.8$, $p<0.0001$	
Step Time Symmetry	$H(2) = 0.25$, $p=0.88$	
**Patient 3 (N = 77)**		
	Left	*Right*
Step Length	$H(2) = 11.5$, $p<0.05$	$H(2) = 21.7$, $p<0.0001$
Step Time	$H(2) = 3.43$, $p=0.18$	$H(2) = 31.0$, $p<0.0001$
	Combined	
Step Length Symmetry	$H(2) = 24.2$, $p<0.0001$	
Step Time Symmetry	$H(2) = 13.1$, $p<0.05$	
**Patient 4 (N = 150)**		
	Left	*Right*
Step Length	$H(2) = 3.06$, $p=0.22$	$H(2) = 25.6$, $p<0.0001$
Step Time	$H(2) = 0.98$, $p=0.61$	$H(2) = 47.0$, $p<0.0001$
	Combined	
Step Length Symmetry	$H(2) = 13.3$, $p<0.05$	
Step Time Symmetry	$H(2) = 0.125$, $p = 0.94$	

Supplementary Table 6: Post-hoc Dunn's test statistics with Holm-Bonferroni p-value adjustment for in-clinic gait metrics comparison during Phase 3 of the study (**Extended Data Fig. 3**, double-blind aDBS testing). Comparison were performed across clinically optimized DBS (cDBS), Ramp-Up aDBS (RU-aDBS), and Ramp-Down aDBS (RD-aDBS).

**Dunn's Test Sample Sizes and Statistics**			
	cDBS	RU-aDBS	RD-aDBS
Patient 2	66	70	81
Patient 3	19	29	29
Patient 4	51	32	67

**Comparisons**						
	**cDBS – RU-aDBS**		**cDBS – RD-aDBS**		**RU-aDBS – RD-aDBS**	
Patient 2	Left	Right	Left	Right	Left	Right
Step Length	-	z = -3.75 (*p<0.001*)	-	*z* = -0.52 (*p*=0.60)	-	*z* = 3.42 (*p<0.05*)
Step Time	*z* = 0.96 (*p*=0.34)	-	*z* = 2.72 (*p<0.05)*	-	*z* = 1.75 (*p*=0.16)	-
Step Length Symmetry	*z* = -3.26 (*p*<0.05)		*z* = -4.92 (*p*<0.0001)		*z* = -1.57 (*p*=0.11)	
Step Time Symmetry	-		-		-	
Patient 3	Left	Right	Left	Right	Left	Right
Step Length	*z* = -3.30 (*p<0.05*)	*z* = 4.57 (*p<0.0001*)	*z* = -2.60 (*p<0.05*)	*z* = 3.47 (*p<0.05*)	*z* = 0.79 (*p*=0.43)	*z* = -1.24 (*p*=0.21)
Step Time	-	*z* = 5.41 (*p*<0.0001)	-	*z* = 4.32 (*p*<0.0001)	-	*z* = -1.23 (*p*=0.22)
Step Length Symmetry	*z* = -1.79 (*p*=0.07)		*z* = -4.73 (*p*<0.0001)		*z* = -3.31 (*p<0.05*)	
Step Time Symmetry	*z* = -2.08 (*p*=0.08)		*z* = -3.62 (*p*<0.001)		*z* = -1.73 (*p*=0.8)	
Patient 4	Left	Right	Left	Right	Left	Right
Step Length	-	*z* = 0.15 (*p*=0.88)	-	*z* = 4.54 (*p*<0.0001)	-	*z* = 3.77 (*p<0.001*)
Step Time	-	*z* = 1.91 (*p*=0.06)	-	*z* = 6.71 (*p*<0.0001)	-	*z* = 1.42 (*p*<0.001)
Step Length Symmetry	*z* = 0.67 (*p*=0.50)		*z* = 3.48 (*p<0.05*)		*z* = 2.31 (*p*<0.05)	
Step Time Symmetry	-		-		-	
