# Supplementary material for: Adaptive deep brain stimulation for dynamic gait control in Parkinson’s disease: a randomized feasibility trial
Source: Nat Med. 2026 Jun 15;32(8):2803–14. doi: 10.1038/s41591-026-04434-2 (PMC13473014; doi:10.1038/s41591-026-04434-2)
Supplement: Supplementary file 2 [file 41591_2026_4434_MOESM2_ESM.pdf]

nature portfolio

Corresponding author(s): Doris D. Wang

Last updated by author(s): Apr 21, 2026

Reporting Summary

Nature Portfolio wishes to improve the reproducibility of the work that we publish. This form provides structure for consistency and transparency in reporting. For further information on Nature Portfolio policies, see our Editorial Policies and the Editorial Policy Checklist.

Statistics

For all statistical analyses, confirm that the following items are present in the figure legend, table legend, main text, or Methods section.

n/a	Confirmed	
☐	☒	The exact sample size (n) for each experimental group/condition, given as a discrete number and unit of measurement
☐	☒	A statement on whether measurements were taken from distinct samples or whether the same sample was measured repeatedly
☐	☒	The statistical test(s) used AND whether they are one- or two-sided<br>*Only common tests should be described solely by name; describe more complex techniques in the Methods section.*
☐	☒	A description of all covariates tested
☐	☒	A description of any assumptions or corrections, such as tests of normality and adjustment for multiple comparisons
☐	☒	A full description of the statistical parameters including central tendency (e.g. means) or other basic estimates (e.g. regression coefficient) AND variation (e.g. standard deviation) or associated estimates of uncertainty (e.g. confidence intervals)
☐	☒	For null hypothesis testing, the test statistic (e.g. F, t, r) with confidence intervals, effect sizes, degrees of freedom and P value noted<br>*Give P values as exact values whenever suitable.*
☒	☐	For Bayesian analysis, information on the choice of priors and Markov chain Monte Carlo settings
☒	☐	For hierarchical and complex designs, identification of the appropriate level for tests and full reporting of outcomes
☐	☒	Estimates of effect sizes (e.g. Cohen's d, Pearson's r), indicating how they were calculated

Our web collection on statistics for biologists contains articles on many of the points above.

Software and code

Policy information about availability of computer code

Data collection

Neural data was collected from 5 patients with the Medtronic Summit RC+S DBS device paired with a custom patient facing graphical user interface (https://github.com/openmind-consortium/App-SCBS-PatientFacingApp).

Behavioral data was collected using two systems: Delsys Trigno/Centro and Xsens Awinda. The associated softwares with these systems were used to pre-process data originating from the respective systems. Data collected during pre- and post-DBS clinical optimization utilized the Delsys Trigno, and was switch to the Centro for double-blind aDBS testing in three patients. Xsens Awinda was used for all stages of data collection. Xsens data was used to calculate spatial gait metrics, while the Delsys data was used for data synchronization between the neural and Xsens data streams, as well as temporal gait metrics.

At-home gait monitoring data was collected using the Rover wareable device. The data was uploaded to the company's proprietary cloud software to determine gait metrics.

Patients 2, 3, and 4 submitted online motor diaries during double-blind randomized testing comparing continuous DBS and adaptive DBS.

Data analysis

Open source – R libraries
R base 4.5.0
tidyverse 2.0.0
cowplot 1.1.3
export 0.3.0
rstatix 0.7.2
gghalves 0.1.4

lmerTest 3.1-3
ggsignif 0.6.4
emmeans 1.11.0
ordinal 2023.12-4.1
FSA 0.10.0

Open source – Python libraries
Python version 3.10.8
numpy 1.23.4
pandas 1.5.0

Commercial software – MATLAB 2020a (The MathWorks Inc., Natick, MA)
Custom code: We used custom code to preprocess raw JSON neural data (https://github.com/openmind-consortium/Analysis-rcs-data), convert the processed neural data to simulated on-board power values (https://github.com/Weill-Neurohub-OPTiMaL/rcs-simulation), align the processed neural, Delsys, and Xsens data streams, identify gait phase biomarkers, and calculate temporal and spatial gait metrics from the processed Delsys and Xsens data. Relevant code to reproduce and generate the main findings of this study will be made available at https://github.com/UCSF-wang-lab/FaDBS within one month of publication.

For manuscripts utilizing custom algorithms or software that are central to the research but not yet described in published literature, software must be made available to editors and reviewers. We strongly encourage code deposition in a community repository (e.g. GitHub). See the Nature Portfolio guidelines for submitting code & software for further information.

Data

Policy information about availability of data

All manuscripts must include a data availability statement. This statement should provide the following information, where applicable:

- Accession codes, unique identifiers, or web links for publicly available datasets
- A description of any restrictions on data availability
- For clinical datasets or third party data, please ensure that the statement adheres to our policy

Due to the inclusion of identifiable clinical and neurophysiological data from human participants, data are subject to controlled access to protect participant privacy and comply with institutional and regulatory requirements. Requests for access should be directed to the corresponding author and will be reviewed within a reasonable timeframe (typically within 2–4 weeks), contingent on institutional approvals and data use agreements that restrict re-identification and redistribution. Requests will be reviewed based on scientific merit, ethical review, and available resources. Following approved requests, de-identified individual patient data will be made available for reuse. The source data used to generate the figures in this paper is provided and will be stored in a Data Archive for the BRAIN Initiative (DABI) database. Link will be updated once the database is approved.

Research involving human participants, their data, or biological material

Policy information about studies with human participants or human data. See also policy information about sex, gender (identity/presentation), and sexual orientation and race, ethnicity and racism.

Reporting on sex and gender

Four patients were male and one was female. Sex and gender effects were not analyzed in this study.

Reporting on race, ethnicity, or other socially relevant groupings

NA

Population characteristics

The study population consisted of five patients with Parkinson's disease and gait impairments. The four male patients were aged 53–68 years, with disease durations of 8–21 years and off/on-medication MDS-UPDRS III scores of 31–42/16–19. The female patient was 68 years old, with a disease duration of 4 years and off/on-medication MDS-UPDRS III scores of 21/9. All patients underwent deep brain stimulation surgery at the University of California, San Francisco, and received depth electrodes in the internal segment of the globus pallidus (Medtronic model 3387). Each patient was also implanted with a chronic electrocorticography strip (Medtronic model 0913025) for neural recording only.

Gait impairments varied across the cohort: two patients (Patients 2 and 5) presented with freezing, festination, and postural imbalance; one patient (Patient 3) had festination and postural imbalance that progressed to freezing; one patient (Patient 1) had postural imbalance; and one patient (Patient 4) exhibited leg dragging.

Recruitment

Prior to enrollment, patients were recruited from a specialized movement disorders and neurosurgery program at a tertiary academic medical center at the University of California, San Francisco. Eligible participants were individuals with Parkinson's disease undergoing deep brain stimulation implantation who consented to participate in research involving neural recordings and adaptive stimulation. This recruitment strategy may introduce selection bias toward patients who are highly motivated, medically suitable for DBS, and willing to participate in research protocols, which may limit generalizability. However, the within-subject crossover design mitigates inter-individual variability and supports internal validity of the findings.

To be eligible for enrollment, patients were required to meet specific clinical criteria.
Inclusion criteria were age 18-75 years, the ability to give informed consent, and the ability to comply with study follow-up visits for brain recording, testing of adaptive stimulation, and clinical assessments. Patients were required have been diagnosed with idiopathic PD for at least three years, undergone appropriate therapy with oral medications with inadequate relief as determined by a movement disorders neurologist, have gait impairments (slowed gait, shuffling steps, postural

	instability, or freezing of gait off medication), have an MDS-UPDRS-III OFF medication score between 20-80 with an improvement of at least 30% when on antiparkinsonian medication, and had a Montreal Cognitive Assessment score ≥ 21 (Table 1). We excluded patients with untreated depression (BDI-II score > 20), a history of suicidal attempts or active suicidal ideation (Yes to #2-5 on C-SSRS), personality or mood symptoms where the study personnel believe will interfere with study requirements, or drug or alcohol abuse. Patients were also excluded if they had a previous cranial surgery, have an implanted stimulation system (e.g. pacemaker), or if they require electroconvulsive therapy, repetitive transcranial magnetic stimulation, or diathermy.
Ethics oversight	The study protocol was approved by USFDA and UCSF IRB (20-32847). The study was registered on ClinicalTrials.gov (NCT04675398).

Note that full information on the approval of the study protocol must also be provided in the manuscript.

Field-specific reporting

Please select the one below that is the best fit for your research. If you are not sure, read the appropriate sections before making your selection.

☒ Life sciences ☐ Behavioural & social sciences ☐ Ecological, evolutionary & environmental sciences

For a reference copy of the document with all sections, see nature.com/documents/nr-reporting-summary-flat.pdf

Life sciences study design

All studies must disclose on these points even when the disclosure is negative.

Sample size	Five patients with Parkinson's disease were included in this study. One patient had a single implant in the left hemisphere, while the other four were implanted bilaterally. All patients received aDBS, resulting in data from nine hemispheres. The amount of data collected from each patient depended on the adaptive parameter optimization phase, with some patients requiring more visits than others. Additionally, not all five patients underwent double-blind testing, which further increased the amount of data from those who did. The number of in-clinic days ranged from 4 to 17, with multiple recordings obtained on each day.<br><br>Sample size (n = 5) was determined based on feasibility considerations for an early-stage, first-in-human study involving invasive neural recordings and adaptive stimulation. No formal power calculation was performed. The sample size is consistent with prior studies using implanted bidirectional neurostimulation systems and is sufficient to demonstrate feasibility, safety, and preliminary efficacy signals, as well as to enable within-subject comparisons across stimulation conditions.
Data exclusions	Outliers within different gait metrics were excluded from analysis according to the interquartile range method, which classifiers a data point as an outlier if it is not within Q1-1.5*IQR to Q3+1.5*IQR. Turns during overground walking were excluded, except for Extended Data Fig. 3 which specifically analyzed turning gait metrics, as this study focused on straight-line overground walking.
Replication	No explicit attempt at replicating the experimental findings was performed. This study is a prospective, within-subject, randomized crossover clinical feasibility study in human participants. Traditional biological replication is not directly applicable, as each participant serves as their own control across stimulation conditions. Multiple repeated measures were collected within each participant across conditions to ensure reliability of observed effects.
Randomization	All patients received ramp-up aDBS, while only three patients received ramp-down aDBS. This discrepancy was due to constraints with the other patients: Patient 1 lived far from our site, and Patient 5 became wheelchair-bound. For patients in the double-blind testing phase, the three stimulation conditions were randomized, with each condition lasting 8–10 days. Randomization sequences were generated using a custom MATLAB script by J.H.M. prior to the start of the double-blind testing phase.
Blinding	Participants and outcome assessors were blinded to stimulation condition during the randomized crossover portion of the study. Stimulation programs were configured by investigators not involved in outcome assessment. In portions of the study involving real-time algorithm development and validation, blinding was not feasible due to the need for active parameter adjustment; however, objective kinematic and neurophysiological metrics were used to minimize bias. K.H.L was unblinded during data analysis at the conclusion of the study protocol.

Reporting for specific materials, systems and methods

We require information from authors about some types of materials, experimental systems and methods used in many studies. Here, indicate whether each material, system or method listed is relevant to your study. If you are not sure if a list item applies to your research, read the appropriate section before selecting a response.

Materials & experimental systems

n/a	Involved in the study
☒	☐ Antibodies
☒	☐ Eukaryotic cell lines
☒	☐ Palaeontology and archaeology
☒	☐ Animals and other organisms
☐	☒ Clinical data
☒	☐ Dual use research of concern
☒	☐ Plants

Methods

n/a	Involved in the study
☒	☐ ChIP-seq
☒	☐ Flow cytometry
☐	☒ MRI-based neuroimaging

Clinical data

Policy information about clinical studies

All manuscripts should comply with the ICMJE guidelines for publication of clinical research and a completed CONSORT checklist must be included with all submissions.

Clinical trial registration: NCT04675398

Study protocol: A description of the study can be found at our clinicaltrials.gov website: https://clinicaltrials.gov/study/NCT04675398.

Data collection: Data for this study were collected between December 2021 and February 2025. In-clinic data were obtained in a dedicated testing room within the Departments of Neurology and Neurological Surgery at the University of California, San Francisco. At-home gait data were collected in patients' homes and within the community during their everyday activities.

Outcomes: This clinical trial is a single-center, phase 1 early feasibility study designed to develop and evaluate the effects of a novel gait-phase–synchronized adaptive stimulation therapy compared with clinically optimized continuous deep brain stimulation. The primary outcome were: (1) successful identification of gait phase biomarkers to implement aDBS and (2) accuracy of contralateral leg swing biomarkers in real-time adaptive DBS control. Our primary clinical outcome was changes in gait metrics. Our secondary outcome was changes in other Parkinson's disease symptoms to ensure they were not adversely affected by the aDBS therapy. These measures were obtained at home using a Rover device (Sensoplex Inc.) to calculate gait metrics, in clinic using our own data collection equipment, and through a digital diary that patients in the double-blind section were instructed to complete.

Plants

Seed stocks: NA

Novel plant genotypes: NA

Authentication: NA

Magnetic resonance imaging

Experimental design

Design type: Structural imaging only

Design specifications: Structural imaging only

Behavioral performance measures: Structural imaging only

Acquisition

Imaging type(s): Structural imaging only

Field strength: 3T

Sequence & imaging parameters: Volumetric T1-weighted gradient echo without contrast (GE MRI 3D T1 BRAVO). Axial T2-weighted fast spin echo, interleaved.

Area of acquisition: Whole brain.

Diffusion MRI: ☐ Used ☒ Not used

Preprocessing

Preprocessing software: The Locate Electrodes Graphical User Interface Toolbox (version 1.2) and Lead-DBS Toolbox (version 2.6)

Normalization: Electrode reconstruction used post-implantation high resolution CT images that were coregistered to preoperative T1-weighted 3T MRI using a rigid, linear affine transformation. Coregistration accuracy was verified by visual inspection and, when necessary, refined using an additional brain shift correction routine to align subcortical anatomy. Then, electrode artifacts were identified on the CT images and fit to know electrode geometry. Cortical electrodes were additionally projected to the MRI-rendered pial surface. Lastly, electrode locations were normalized into Montreal Neurological Institute space and visualized using the ICBM152 atlas for the depth electrodes and DISTAL atlas for the electrocorticography leads.

Normalization template: Electrode locations were normalized into Montreal Neurological Institute space. Depth electrodes were visualized using the DISTAL atlas and the electrocorticography lead using the ICBM152 atlas.

Noise and artifact removal: The Locate Electrodes Graphical User Interface Toolbox (version 1.2) and Lead-DBS Toolbox (version 2.6)

Volume censoring: NA

Statistical modeling & inference

Model type and settings: NA

Effect(s) tested: NA

Specify type of analysis: ☒ Whole brain ☐ ROI-based ☐ Both

Statistic type for inference (See Eklund et al. 2016): Imaging was only used to record depth lead and electrocorticography strip location.

Correction: NA

Models & analysis

n/a	Involved in the study	
☒	☐	Functional and/or effective connectivity
☒	☐	Graph analysis
☒	☐	Multivariate modeling or predictive analysis
