# Supplementary material for: Adaptive deep brain stimulation for dynamic gait control in Parkinson’s disease: a randomized feasibility trial
Source: Nat Med. 2026 Jun 15;32(8):2803–14. doi: 10.1038/s41591-026-04434-2 (PMC13473014; doi:10.1038/s41591-026-04434-2)
Supplement: Supplementary file 3 [file 41591_2026_4434_MOESM3_ESM.pdf]

nature portfolio

Peer Review File

Adaptive Deep Brain Stimulation for Dynamic Gait Control in Parkinson's Disease: a randomized feasibility trial

Corresponding Author: Dr Doris Wang

Parts of this Peer Review File have been redacted as indicated to remove third-party material.

This file contains all reviewer reports in order by version, followed by all author rebuttals in order by version.

Version 0:

Reviewer comments:

Reviewer #1

(Remarks to the Author)
The concept of dynamic DBS modulation during gait at this subsecond time scale is highly innovative and novel, based on either a particularly activity or activity phases. In this study in PD patients implanted with Summit RC+S (GP + ECoG cortical paddle). Both LFP and gait data were collected (presumably through external wireless connection to a PC) during walking (in 4-10 sessions), using gait digitization in the laboratory. Based on these wirelessly recording data a control signal LFP was identified related to contralateral leg swing (not clear if recording from GP contacts, cortical contacts, or both, from one hemisphere). Based on these data from the gait lab while directly streaming the identified LFP signals to a PC. It is not clear if both hemispheres were optimized or just one.
A comprehensive search (presumably in the lab with direct streaming of LFPs and gait monitoring) was conducted for specific, optimal LFP biomarkers: GP in 4 hemispheres, PM in 3, M1 and S1 in 1 each. Ramp rate controls the speed of changes, > 1.9 mA per 400 ms, and amplifier blanking set to 1 ms. The changes in FFT led to a binary classifier during the specific, identified gait phase.
Once the relevant LFP frequency bandwidth was detected and optimized then this bandwidth was embedded into the internal control of the Summit RC+S, for at-home ramping between 0.5 and 1.0 X of the prior clinically determined DBS amplitude. An accelerometer was attached to each leg to monitor gait at home.
In results the M1 paddle contacts was identified in 8/9 hemispheres, less so GP (5/9) and in 3 pts GP was unhelpful. Presumably the LFP result was the sole measure of gait phase or whether the patient was walking, rather than identify (using for example, the external or internal accelerometers) whether the patient was actually walking. This assumes that the LFP changes would not be triggered by, for example, sitting with swinging the legs, which might be a false event. Were all patients undergoing bilateral control? The results show more symmetry and less falls. The overall results are very interesting though but limited to symmetry of gait rather than stride length or overall speed.

Questions:
1) Please clarify how the in lab streaming was conducted – 4 channels at 500 Hz along with the gait digitizer into a PC (ie, external control mode of the RC+S)? What about variable wireless packet times (ie, can vary 100-150 ms, a significant part of the gait period)? Were both IPGs streaming at the same time to the same device? Were the initial FFTs for determining gait phase conducted on the external PC or simply transferred from the IPG to the external PC? Presumably the in lab streaming results are shown in Figs 1, 2, 3 and perhaps 4? Which figures were analyzed from the home trial (ie, just Fig 5)?
2) short FFTs in the embedded mode may be very noisy – how was this controlled (ie, even the 1 sec standard FFT is highly variable compared to a longer 8-10 sec recording)?
3) How did the embedded mode classifier know the patient was walking? How much crossover to other activities where the leg was moving? This could be sitting or if the patient was having dyskinesias with leg movement, for example. Was this assessed through the external accelerometers on the leg or some other state classifier approach?
4) How was the data collected from external, home accelerometers and how were these data combined with the embedded mode RC+S output?
4) How did the authors decide on a range of 0.5 – 1.0 X clinically appropriate DBS? Would it be better to turn down the device to a lower value (ie, 0)?
5) Were other phases of gait identified to have their own unique marker as well?
6) What is the range of stim currents as clinically determined (could be shown in Fig. 4)?

Overall, a very interesting and novel study but some details could be clarified.

(Remarks on code availability)

Reviewer #2

(Remarks to the Author)
Authors performed a 2-phase (in clinic and at home) double-blind study in 5 patients with Parkinson's disease (one study only conducted in 3 subjects) testing whether gait-phase synchronized adaptive DBS (aDBS) dynamically modulating amplitude during contralateral leg swing was superior to standard continuous DBS (cDBS). Patients carried GPi leads as well as paddles over M1 (one patient only unilaterally). The data seem to support that aDBS is superior to cDBS.

This study is interesting from a technical standpoint and timely as it addresses an unmet need of DBS for PD.

I have the following comments:
1. Authors argue that overstimulation leads to hyperkinesia but miss to acknowledge that most of this literature – as well as gait-related dysfunction – comes from STN DBS. GPi DBS is actually better for dyskinesias when enough stimulation is used.
2. State-dependent LFP fluctuations have been observed during gait with deep electrodes, Authors should better clarify the added value of M1 recording
3. The inclusion of a unilateral patient – and the overall small sample – is an important limitation, also considering that gait tends to be considered a 'bilateral/midline' problem
4. Authors used 'patient-specific neural data' to developed aDBS but it's not clear how many permutations were used, for example, what if aDBS was triggered by ipsilateral swing? Or contralateral heel strike?
5. The study fails in terms of clinical assessments, motor scores are not clear as well as "number of falls and number of freezing episodes", which is left to patient's diaries. This approach is heavily criticized by current literature.
6. "M1 exhibited the highest proportion of hemispheres with at least one discriminative canonical frequency band (8/9), followed by the pallidum (5/9), PM (3/9), and S1 (2/9) (Extended Data Fig. 1). In three patients, pallidal recordings did not result in any frequency bands capable of distinguishing swing phases": I'm confused because 5/9 patients for the pallidum means that at least 3 patients had good distinguishing bands.
7. The issue of turning and how the control signal behaved needs to be discussed. Turning is by definition asymmetrical, with one leg in swing, the other in stance.
8. Why were rump up and rump down chosen? Any individual superiority of one over the other? In particular, ramp up makes sense in patients without side effects of DBS while the opposite in patients with e.g. cortico-spinal spreading of the VTA.
9. Linked to previous comment, the prokinetic effect of GPi is less obvious and I'm not sure how ramping up can result in an immediate benefit of step length.
10. Clinical data are presented in a less intuitive way, probably due to the lack of statistical differences among groups. For example, "at the group level, RU-aDBS significantly increased the odds of reporting fewer falls compared to cDBS (odds ratio [OR] = 4.35)" does it mean that the number of falls were significantly less in the aDBS group?
11. Were near-falls addressed?
12. "No group-level changes were detected for freezing episodes": how about wearable data? Overall this points to a less impactful effect of aDBS. To this point, Authors' claims in the discussion about the efficacy might not be concordant with Results.
13. "Odds of improvement in rigidity were low under both strategies (RUaDBS: OR = 0.114; RD-aDBS: OR = 0.038)": evaluated how? At home?
14. Videos of patients would be of interested.
15. Authors have recently published papers from the same cohort, how are these studies blending with each other?
16. What's the role and effect of L-dopa?
17. "Coordinated reset stimulation, which is designed to desynchronize pathological synchrony, requires precise temporal delivery and has been associated with motor improvements": overall most DBS experts are skeptical that this is effective.
18. "Patient 1, who exhibited severe dyskinesia, showed no improvements in gait metrics, possibly due to the chronic instability of stimulation settings and limited acute testing duration": this is mentioned for the first time in the discussion
19. What's the meaning of "-/-" in table 1?
20. Why was LOW used? This is 'low medication state defined as the same period between the end of their current medication cycle to the next'. It seems quite convoluted to me, was it the 'off' for all conditions?

(Remarks on code availability)

Version 1:

Reviewer comments:

Reviewer #1

(Remarks to the Author)
The authors have significantly revised and clarified the manuscript according to the reviewer comments, particularly that

both cortical M1 and pallidal recording contacts are critical to develop the LFP based changes. They have also clarified the extensive time analysis (ie, > 30 hr per patient) needed to derive these specific results, which will clearly require significant revision before being applicable to a larger cohort. All of the prior questions have been answered suitably in the comments and manuscript, particularly the details, methods and limitations of the study.

(Remarks on code availability)

Reviewer #2

(Remarks to the Author)
I thank the Authors for editing the manuscript.
I have the following comments:
1. “While less common than in the STN, this effect is well-documented in the GPi, particularly with ventral stimulation”: although possible, ventral GPI is mainly anti-kinetic, the dorsal stimulation of the nucleus can cause dyskinesias. In any case, these are rare occurrences and almost never disabling
2. “We have updated the text in the results to clarify comparisons are being made with reported values as opposed to objective measures. Line 238-243”: reporting clinical effects in terms of ORs remains odd.
3. “rigidity was evaluated at home via patient self-reporting”: no PD experts would agree that this is feasible, as rigidity is by definition assessed by the examiner’s act of moving a body part. In fact, very rarely a PD patient would complain of ‘rigidity’ or ‘stiffness’.
4. “Videos are taken from our IMU based motion capturing system (Xsens) to protect our patient’s identity”: this is odd and in contrast with the vast majority of clinical studies, where real videos are presented.
5. Overall, I still don’t grasp whether this new model of stimulation is superior to the already approved aDBS merely based on pallidal beta.

(Remarks on code availability)

Open Access This Peer Review File is licensed under a Creative Commons Attribution 4.0 International License, which permits use, sharing, adaptation, distribution and reproduction in any medium or format, as long as you give appropriate credit to the original author(s) and the source, provide a link to the Creative Commons license, and indicate if changes were made.
In cases where reviewers are anonymous, credit should be given to 'Anonymous Referee' and the source.
The images or other third party material in this Peer Review File are included in the article’s Creative Commons license, unless indicated otherwise in a credit line to the material. If material is not included in the article’s Creative Commons license and your intended use is not permitted by statutory regulation or exceeds the permitted use, you will need to obtain permission directly from the copyright holder.
To view a copy of this license, visit https://creativecommons.org/licenses/by/4.0/

We greatly appreciate the insightful comments from the reviewers and have responded to each comment below. We hope these responses not only offer clarity for our study but also strengthen our manuscript.

Reviewer #1 comment response:

The following three questions were presented in their summary, which we are taking the opportunity to answer first.

- **Question:** "It is not clear if both hemispheres were optimized or just one."

  Response: We thank the reviewer for this important observation. All implanted hemispheres underwent optimization of the biomarker and adaptive parameters of the neurostimulator.

  Change: We have updated the third paragraph in the Results – Patients overview and study design section to indicate all work was done per implanted hemisphere. Additionally, we added text to explicitly say Patients 1-3 and 5 were bilateral and Patient 4 was unilateral. Changes are found on lines 115-120.

  > "Control signals were derived from single-frequency band power at one recording site per implanted hemisphere, identified through a data-driven search... Optimal neural features were embedded into the Summit RC+S device for each implanted hemisphere (bilaterally for Patients 1-3 and 5, and unilaterally for Patient 4) and adaptive parameters were manually refined to maximize detection accuracy."

- **Question:** "Presumably the LFP result was the sole measure of gait phase or whether the patient was walking, rather than identify (using for example, the external or internal accelerometers) whether the patient was actually walking. This assumes that the LFP changes would not be triggered by, for example, sitting with swinging the legs, which might be a false event."

  Response: The reviewer is correct in that we used LFP to identify gait phase during walking rather than relying on external or internal accelerometers, as the accelerometer signals cannot be used in the RC+S on-board LDA classifier to trigger aDBS. As to whether our biomarkers can be falsely triggered by leg swing or arm movements, or the specificity of our LDA, we spent many hours optimizing the classifier to confirm that the biomarker and spectral power threshold was

sufficiently specific to the gait cycle. False-positive stimulation changes occurred infrequently during sitting with arm or leg movements but were not time-locked to a specific movement. A more detailed explanation is provided to the official questions 3 below.

We provided two videos, one showing a patient with right foot dyskinesia (Patient 1) and another showing a patient seated while moving their arms and hands.

Change: We have now included the number of optimization visits in Methods section (Line 1045-1047) and added the videos as Supplementary Videos 2 and 3.

> "The optimization process required a mean of 6.4 visits per patient (range: 1–10), with each visit lasting approximately 5 hours."

- **Question:** "Were all patients undergoing bilateral control?"

  Response: We thank the reviewer for giving us a chance to clarify this detail. Patients 1-3 and 5 were under bilateral control, while Patient 4 was unilateral.

  Change: See our changes to Question 1. Briefly, we have altered the text to explicitly say which patients are under bilateral vs unilateral control.

The following questions are from the official question list.

1. **Question:** "Please clarify how the in lab streaming was conducted – 4 channels at 500 Hz along with the gait digitizer into a PC (ie, external control mode of the RC+S)? What about variable wireless packet times (ie, can vary 100-150 ms, a significant part of the gait period)? Were both IPGs streaming at the same time to the same device? Were the initial FFTs for determining gait phase conducted on the external PC or simply transferred from the IPG to the external PC? Presumably the in lab streaming results are shown in Figs 1, 2, 3 and perhaps 4? Which figures were analyzed from the home trial (ie, just Fig 5)?"

   Response: We thank the reviewer for this detailed inquiry regarding the technical implementation. We appreciate the opportunity to clarify the distinction between the embedded control loop and the data telemetry. We will address this question in pieces.

   a) <u>Embedded vs. External Control (Latency)</u>: The reviewer is correct that wireless packet transmission to the PC involves variable latency (100–150 ms). However, this study utilized fully embedded closed-loop control. The neural stimulator calculates

the FFT and modulates the stimulation amplitude locally on the device's internal processor. Consequently, the wireless telemetry latency applies only to the data transmission to the PC and does not affect the timing of the stimulation delivery, which occurs in real-time on the IPG.

b) Data Buffering & Timestamps: As described in Sellers et al. (2021), https://doi.org/10.3389/fnhum.2021.714256, the IPG buffers sensed neural data and FFT power values in its internal memory with precise hardware timestamps at the time of acquisition. When a sufficient packet size is reached (~50 samples), the data are streamed to the PC. The open source data processing packaged (found here: https://github.com/openmind-consortium/Analysis-rcs-data) reconstructs the time-series based on the internal device timestamps, not the packet arrival time, ensuring temporal accuracy for offline analysis.

c) Bilateral Streaming: For bilaterally implanted patients, both IPGs streamed data to the host PC simultaneously, as validated in Gilron et al. (2021).

d) Synchronization between the neural data and kinematic data : In-clinic data collection involved synchronizing the neural stream with external kinematic data. We used a setup where the external systems (motion capture, force sensitive resistors, and external accelerometry) were hardware-triggered. These external data were then synchronized with the neural recordings offline by aligning the signal from the IPG's integrated accelerometer (located over the pectoralis under the clavicles) with the external accelerometer devices placed over the chest.

e) **Figure Data Sources**: We confirm that Figures 1, 2, 3, and 4 used in-lab streaming data, while figure 5 did not.

2. **Question:** "short FFTs in the embedded mode may be very noisy – how was this controlled (ie, even the 1 sec standard FFT is highly variable compared to a longer 8-10 sec recording)?"

   Response: We thank the reviewer for highlighting this trade-off. We acknowledge that using short FFT windows increases spectral variance (noise). However, capturing sub-second gait phase modulations require high temporal resolution. Calculating the FFT with windows of longer durations (e.g., 8–10 s) would smooth out the transient signal changes necessary to drive the aDBS algorithm. Furthermore, the hardware imposes a strict technical constraint: the embedded signal processing buffer is limited to 1024 samples. At our sampling rate of 500 Hz, this precludes FFT calculations over windows longer than ~2 seconds. To ensure

that the resulting spectral variance did not compromise control accuracy, we performed extensive in-clinic validation (up to 10 visits per patient, 3–4 hours per session), visually monitoring detector performance in real-time and verifying signal quality offline.

3. **Question:** “How did the embedded mode classifier know the patient was walking? How much crossover to other activities where the leg was moving? This could be sitting or if the patient was having dyskinesias with leg movement, for example. Was this assessed through the external accelerometers on the leg or some other state classifier approach?”

   Response: We thank the reviewer for this important question regarding classifier specificity. The embedded system operated as a direct phase-detector rather than a hierarchical state-machine (i.e., it did not separately detect "Walking" then "Swing"). Specificity was achieved by including periods of rest and sitting with arm and leg movements into our data-driven biomarker search. The search selected the frequency band that maximized the separation between "Contralateral Swing" and all other states, including these non-gait movements. Regarding the assessment method, we relied on extensive in-clinic validation (average 6 visits, ~5 hours each) using visual verification and offline analysis of the stimulator state and multiple external sensors, rather than external accelerometers alone. We confirmed that the biomarker and spectral power threshold was sufficiently specific to the gait cycle. False-positives did occur while the patient was sitting with arm and leg movements, but they were infrequent and not time-locked to any specific movement. Additionally, Patient 1 who experienced dyskinesia on cDBS also displayed dyskinesia on aDBS. However, the classifier output remained stable while they were seated with head and leg movements. Similarly, Patient 2-5, who did not display any dyskinesia, did engage in voluntary non-gait movements (e.g., fidgeting of arms and legs), which the classifier successfully rejected. Lastly, we refer the reviewer to Supplementary Video 2 and 3, which demonstrates the classifier output remaining stable while the patient is sitting and engaging in non-gait movements.

   Change: We have updated the text in the methods section to include that periods of rest were considered during the biomarker search and how many hours of optimization. Lines 1005-1006.

   > “To ensure detector specificity, the simulation dataset explicitly included periods of rest with and without arm and leg movements.”

We have updated the text in the results section to include that false-positives did occur but were infrequent and not time-locked to any specific movement. Lines 172-174.

> "False positives during non-gait activities (e.g., sitting with arm/leg movement) were infrequent and not time-locked to specific movements (see Supplementary Videos 2 and 3)."

We have updated the limitations paragraph to include dyskinesia. Lines 401-404.

> "The classifier successfully rejected non-gait movements, including voluntary limb movements in Patients 2–5 and dyskinetic episodes in Patient 1. However, future work is needed to validate classifier performance in a larger cohort with severe, distinct dyskinetic patterns."

4. **Question:** "How was the data collected from external, home accelerometers and how were these data combined with the embedded mode RC+S output?"

   Response: We thank the reviewer for this inquiry. The external home monitoring utilized the Rover system, consisting of bilateral ankle accelerometers that passively collect data after placement. These data are uploaded to the manufacturer's cloud platform for processing and analysis. Importantly, this dataset was not synchronized or combined with the embedded RC+S neural output. This dataset served exclusively as the source for behavioral gait metrics during the double-blinded crossover phase (Patients 2–4).

   Change: We have updated the text in the last paragraph of the Results – Patients overview and study design section to reflect that the data was collected passively and uploaded to the manufacturer cloud platform for quantification of gait metrics at home. Lines 127-130.

   > "Additionally, patients wore a tri-axial accelerometer (Rover, Sensoplex Inc., Redwood City, CA) that passively collect data, which were then uploaded to the manufacturer's cloud platform for data processing and quantification of at-home gait metrics.

5. **Question:** "How did the authors decide on a range of 0.5 – 1.0 X clinically appropriate DBS? Would it be better to turn down the device to a lower value (ie, 0)?"

Response: We thank the reviewer for this inquiry. The 0.5–1.0x amplitude range was selected based on prior evidence (Louie et al., 2021) and safety considerations. While Louie et al. tested a wider range (0–1.0x), they observed limited efficacy using external, predictive timing of stimulation delivery in the off-medication state. In contrast, our approach utilizes real-time, embedded sensing to deliver stimulation in the on-medication state. Clinically, we established the 0.5x lower bound to ensure safety: this floor prevents the undertreatment of other cardinal motor symptoms (e.g., tremor/rigidity) during the low-amplitude phase, maintaining general motor control during continuous daily use.

Change: We have updated text in the Methods – Adaptive deep brain stimulation section to clarify how this range was selected, and a new reference was added. Changes can be found on line 983-986.

> **"**The stimulation amplitude ranged from 0.5x to 1.0x the patient's clinically optimized therapeutic value. This range was selected to maintain a continuous therapeutic baseline for other cardinal motor symptoms during low-amplitude phases, as prior research indicated limited efficacy with a broader 0-1.0x range [Louie et. al., 2021]."

6. **Question:** "Were other phases of gait identified to have their own unique marker as well?"

   Response: We thank the reviewer for this interesting question. In our exploratory analysis, we indeed observed neural modulations locked to other phases of the gait cycle (e.g., toe-off or double support time) in some individual patients. However, unlike the swing-phase biomarker, these other phase-related signals were not consistently detectable across all patients in our cohort. Given that the primary objective of this study was to establish the feasibility of a generalized framework for aDBS, we prioritized the swing-phase biomarker because it was the only feature identifiable in all five patients. We have chosen to focus the manuscript on this shared feature to provide a clear, unified narrative for clinical translation.

7. **Question:** "What is the range of stim currents as clinically determined (could be shown in Fig. 4)?"

   Response: We thank the reviewer for this request. The clinically optimized stimulation amplitudes ranged from 2.8 to 5.5mA across our patients. We have updated the table in Fig. 2 to include units in the amplitude columns and have

added asterisks to designate the values established during standard clinical care. A footnote is also added to the table.

Change: Below is the image of the table from Fig. 2

		Adaptive Parameters				Other Stimulation Parameters		
Patient		Biomarker Source	Biomarker Band(Hz)	Amplitude Min(mA)	Amplitude Max(mA)*	Contacts*	Frequency(Hz)*	Pulse Width(µs)*
Patient 1	Left	GPi	43-45	2.1	4.2	C+2-	140	80
	Right	PM	18-35	1.9	3.8	C+2-	139	80
Patient 2	Left	GPi	6-8	2.5	5.0	C+2-	150	60
	Right	PM	23-31	2.5	5.0	C+1-	150	60
Patient 3	Left	GPi	13-17	2.0	4.0	C+1-	130	60
	Right	GPi	11-17	2.0	4.0	C+1-	130	60
Patient 4	Left	PM	28-29	2.4	4.7	C+1-	130	60
Patient 5	Left	M1	11-13	2.8	5.5	C+1-2-	150	90
	Right	S1	11-12	2.8	5.5	C+1-2-	150	90

*Clinically optimized parameters according to standard clinical care

Reviewer #2 comment response:

1. **Question:** Authors argue that overstimulation leads to hyperkinesia but miss to acknowledge that most of this literature – as well as gait-related dysfunction – comes from STN DBS. GPi DBS is actually better for dyskinesias when enough stimulation is used.

   Response: We thank the reviewer for this critical distinction and for highlighting the clinical nuances between STN and GPi stimulation. We agree that GPi DBS is classically indicated for the suppression of levodopa-induced dyskinesias. However, our reference to "overstimulation" addresses the specific phenomenon of stimulation-induced dyskinesia (distinct from levodopa-induced). While less common than in the STN, this effect is well-documented in the GPi, particularly with ventral stimulation. Studies by Krack et al. (1998), Elkouzi et al. (2019), Lee et al. (2021), and Wong et al. (2025) have all described stimulation-induced dyskinesia following GPi DBS, with the latter analyzing a cohort of 17 patients. More critically, "overstimulation" refers to the loss of neural flexibility due to network entrainment. Work from Cleary et al.(2013) and Olaru et al.(2024) has demonstrated that high-frequency stimulation can entrain pallidal firing, particularly locking neurons into synchronized gamma-band oscillations. Locking the neurons to a specific oscillation may prevent the natural spectral modulation required for effective gait.

Change: We have changed the text in the introduction to more precise with regards to STN and GPi. Lines 58-64.

> "While most of these electrophysiological insights derive from the subthalamic nucleus (STN), parallel dynamics exist in the internal globus pallidus (GPi). During "on" medication states, when dopaminergic therapy effectively controls motor symptoms, continuous DBS may cause overstimulation, leading to adverse effects such as stimulation-induced dyskinesia [krack 1998, elkouzi 2019, lee 2021, and wong 2025]. Furthermore, regarding network dynamics, while neural entrainment to stimulation is often a predictor of improved motor performance, continuous entrainment imposes a static neural state."

2. **Question:** State-dependent LFP fluctuations have been observed during gait with deep electrodes, Authors should better clarify the added value of M1 recording

   Response: We thank the reviewer for this inquiry regarding the specific utility of cortical electrocorticography leads. We found that cortical recordings provided significant added value in two areas: superior classification accuracy and robustness to the stimulation artifact. Empirically, our data-driven search identified the cortical signal as the optimal control feature in 5 of the 9 implanted hemispheres (Fig. 2c). This indicates that relying solely on deep electrodes would have resulted in a suboptimal control signal for a majority of implanted hemispheres. From a technical perspective, sensing from the cortex provides a cleaner signal. The recording site is distant from the stimulation source in the GPi, making the signal less susceptible to stimulation artifacts.

   Change: We have added the following to the discussion. Page 15 of discussion, lines 317-322.

   > "Our findings highlight two key advantages of incorporating cortical signals into adaptive DBS control architectures. First, cortical recordings may provide superior decoding performance, as it provided the optimal control feature in 5 of the 9 implanted hemispheres. Second, cortical electrodes offered substantially cleaner signals during stimulation, increasing robustness and reliability during closed-loop operation."

3. **Question:** The inclusion of a unilateral patient – and the overall small sample – is an important limitation, also considering that gait tends to be considered a 'bilateral/midline' problem

   Response: We thank the reviewer for this critical observation. While we agree that locomotor control has traditionally been conceptualized as a bilateral or midline problem, the clinical expression of gait dysfunction is frequently asymmetric (e.g., unilateral step shortening). Patient 4 provided a unique opportunity to test the mechanistic specificity of our approach. The observation that unilateral aDBS successfully restored step length symmetry demonstrates that the therapy modulates limb-specific dynamics rather than simply inducing global, non-specific modulation. Thus, the inclusion of this patient enhances the study by demonstrating that biomarker-driven aDBS can be personalized to asymmetric clinical presentations.

4. **Question:** Authors used 'patient-specific neural data' to developed aDBS but it's not clear how many permutations were used, for example, what if aDBS was triggered by ipsilateral swing? Or contralateral heel strike?

   Response: We thank the reviewer for this insightful question regarding temporal specificity. In this feasibility study, we focused exclusively on the contralateral swing phase and did not systematically test alternative permutations (e.g., triggering off heel strike) due to the constraints of acute clinical testing and patient fatigue. However, this selection was not arbitrary. Consistent with the established contralateral organization of the motor system, we targeted the hemisphere contralateral to the moving limb to maximize influence. Furthermore, regarding the phase target, stimulating during the stance/heel-strike phase offers limited mechanical opportunity to alter limb movement. Lastly, as detailed in our response to Reviewer #1 (Comment 6), the contralateral swing phase was the only biomarker feature consistently identified across all patients in the study, making it the only viable target for a generalized feasibility framework.

5. **Question:** The study fails in terms of clinical assessments, motor scores are not clear as well as "number of falls and number of freezing episodes", which is left to patient's diaries. This approach is heavily criticized by current literature.

   Response: We thank the reviewer for this critical assessment. We respectfully disagree that the study "fails" on clinical assessment. Rather, we employed a hybrid

approach designed to capture both objective biomechanics and subjective clinical experience. While we acknowledge that patient diaries are subject to recall bias, they remain the regulatory gold standard for assessing "ON-time" and functional independence in Parkinson's disease clinical trials. Furthermore, as noted in Response 11, validated wearable algorithms for the automated detection of falls and freezing in the home setting do not yet exist. Therefore, diaries remain the most reliable method for capturing these specific adverse events. We view the diary data not as a replacement for objective measurement, but as a complementary stream. Diaries reflect the lived experience, while our wearable sensors (Rover) quantify the biomechanical effect (gait metrics). Both are necessary for a comprehensive feasibility profile. To address the reviewer's concern regarding the visibility of standard clinical metrics, we have aggregated the blinded MDS-UPDRS III and Mini-BESTest scores collected during the randomized testing phase (Phase 4) into a dedicated table.

Change: We have added Table 2 to the manuscript, detailing the blinded MDS-UPDRS III and Mini-BESTest scores for Patients 2–4 across the aDBS and cDBS conditions. We have also added a new paragraph in the results section that presents the results of the clinical measurements. Lines 253-263.

> "In addition to patient-reported motor diaries, blinded clinical assessments were administered in-clinic at the end of each testing phase (Table 2). Comparing the three stimulation conditions, both RU- and RD-aDBS strategies generally maintained or improved global motor control relative to cDBS (MDS-UPDRS III). Patients 2 and 3 exhibited improved (lower) motor scores under both aDBS conditions compared to cDBS, with RD-aDBS resulting in the lowest total motor score for Patient 3 (26 vs. 29). Conversely, Patient 4 showed a slight worsening in motor scores under aDBS (+1-3 points). Postural stability scores (Mini-BESTest) improved while on RU-aDBS for Patient 2 (+3 points vs. cDBS), whereas Patient 3 worsened under RU-aDBS. Patient 4 remained stable (21-22 points) across all conditions. Notably, PIGD sub-scores remained largely stable across conditions for Patients 2 and 4, but exhibited a slight improvement (reduction of 1-3 points) for Patient 3."

6. **Question:** "M1 exhibited the highest proportion of hemispheres with at least one discriminative canonical frequency band (8/9), followed by the pallidum (5/9), PM (3/9), and S1 (2/9) (Extended Data Fig. 1). In three patients, pallidal recordings did

not result in any frequency bands capable of distinguishing swing phases": I'm confused because 5/9 patients for the pallidum means that at least 3 patients had good distinguishing bands.

Response: We thank the reviewer for carefully checking these results. We apologize for the confusion caused by the phrasing in the original text. To clarify the breakdown of the 9 implanted hemispheres and whether a canonical frequency band was found in the pallidum: Patient 1 (left and right), Patient 2 (right), Patient 3 (left and right), Patient 4 (none), and Patient 5 (none). The reviewer is correct that phrasing this as "three patients did not result in any frequency bands" was imprecise, as Patient 2 did have a successful band in one hemisphere. We have revised the text to distinguish between patients with total signal failure versus unilateral failure.

Change: We have updated the text in the manuscript to clarify this distinction that we are talking about implanted hemispheres and how one, but not both in a bilaterally implanted patient could have a significant canonical band. Lines 142-144.

> "In two patients (Patients 4 and 5), pallidal recordings did not result in any frequency bands capable of distinguishing swing phases, while Patient 2 exhibited a discriminative band only in the right hemisphere."

7. **Question:** The issue of turning and how the control signal behaved needs to be discussed. Turning is by definition asymmetrical, with one leg in swing, the other in stance.

   Response: We thank the reviewer for raising this important topic. We agree that turns are biomechanically distinct and asymmetrical. Our biomarker search and optimization focused on straight walking to establish a robust baseline for phase-detection and was not explicitly trained on turning mechanics. Regarding the control signal behavior, we observed three primary states during turns: 1) the stimulator continue in the same state until the turn was complete; 2) the state switched once and then continued in the same state until completion; or 3) the stimulation continued to modulate but with a phase delay relative to the swing. Importantly, we retrospectively analyzed turn metrics (steps and it's variability) for Patients 2, 3, and 4 during the double-blind randomization phase to ensure this behavior did not negatively impact gait. Importantly, we retrospectively analyzed turn metrics (step length and variability) for Patients 2, 3, and 4 during the double-blind randomization phase to ensure these behaviors did not negatively impact gait. We found no significant deterioration in turning performance attributable to the adaptive

stimulation. While step length decreased and asymmetry increased during turns (as biomechanically expected), these changes were consistent across both aDBS and cDBS conditions. This indicates that the system remained safe and effective despite the lack of turn-specific optimization.

For transparency, we provide the specific gait metrics during turns in the table below. These data confirm that the observed asymmetry was driven by the turning activity itself rather than the adaptive algorithm.

Changes: We have added this table and figures in Extended Data Table 1 and Extended Data Fig. 3 and added a short description in the results (lines 285-290):

> "Because our study focused on identifying gait-phase biomarkers during straight-line walking, we found there is variable behavior of the state classifier during turning. While step length decreased and asymmetry increased during turns as biomechanically expected (Extended Data Table 1 and Extended Data Fig. 3), these changes were consistent across both aDBS and cDBS conditions, indicating that the system remained safe and effective despite the lack of turn-specific optimization."

Additionally, we have added in the methods text regarding the exclusion of turns from gait metric analysis. Lines 968-969.

> "Turns were excluded from all analysis, as this study focused on straight-line overground walking."

Extended Data Table 1

<table>
<tr><td colspan="2"></td><td colspan="3"><u>Step Length (m)</u></td><td colspan="3"><u>Step Time (s)</u></td></tr>
<tr><td colspan="2"></td><td>μ ± SD</td><td>CV</td><td>Absolute Asymmetry</td><td>μ ± SD</td><td>CV</td><td>Absolute Asymmetry</td></tr>
<tr><td colspan="2">Patient 2</td><td colspan="6"></td></tr>
<tr><td rowspan="2">cDBS</td><td>Left</td><td>0.64±0.07</td><td>0.12</td><td rowspan="2">9.3%</td><td>0.63±0.04</td><td>0.06</td><td rowspan="2">3.2%</td></tr>
<tr><td>Right</td><td>0.65±0.08</td><td>0.12</td><td>0.62±0.05</td><td>0.08</td></tr>
<tr><td rowspan="2">RU-aDBS</td><td>Left</td><td>0.63±0.05</td><td>0.07</td><td rowspan="2">6.9%</td><td>0.64±0.04</td><td>0.06</td><td rowspan="2">3.3%</td></tr>
<tr><td>Right</td><td>0.60±0.07</td><td>0.12</td><td>0.62±0.05</td><td>0.08</td></tr>
<tr><td rowspan="2">RD-aDBS</td><td>Left</td><td>0.62±0.06</td><td>0.10</td><td rowspan="2">5.4%</td><td>0.64±0.02</td><td>0.04</td><td rowspan="2">2.7%</td></tr>
<tr><td>Right</td><td>0.64±0.04</td><td>0.06</td><td>0.63±0.04</td><td>0.07</td></tr>
<tr><td colspan="2">Patient 3</td><td colspan="6"></td></tr>
<tr><td rowspan="2">cDBS</td><td>Left</td><td>0.58±0.09</td><td>0.15</td><td rowspan="2">27.8%</td><td>0.70±0.09</td><td>0.13</td><td rowspan="2">17.0%</td></tr>
<tr><td>Right</td><td>0.33±0.11</td><td>0.32</td><td>0.50±0.06</td><td>0.11</td></tr>
</table>

RU-aDBS	*Left*	0.47±0.12	0.26	19.6%	0.65±0.08	0.13	10.6%
	Right	0.52±0.13	0.25		0.66±0.11	0.16	
RD-aDBS	*Left*	0.49±0.08	0.17	8.6%	0.67±0.07	0.11	7.1%
	Right	0.47±0.09	0.19		0.61±0.06	0.10	
**Patient 4**							
cDBS	*Left*	0.42±0.04	0.09	5.8%	0.50±0.03	0.07	5.0%
	Right	0.44±0.06	0.14		0.46±0.04	0.09	
RU-aDBS	*Left*	0.44±0.04	0.10	6.6%	0.50±0.03	0.06	3.3%
	Right	0.44±0.05	0.11		0.48±0.02	0.05	
RD-aDBS	*Left*	0.42±0.06	0.15	9.9%	0.50±0.03	0.06	1.2%
	Right	0.50±0.06	0.13		0.50±0.03	0.06	

Extended Data Fig. 3

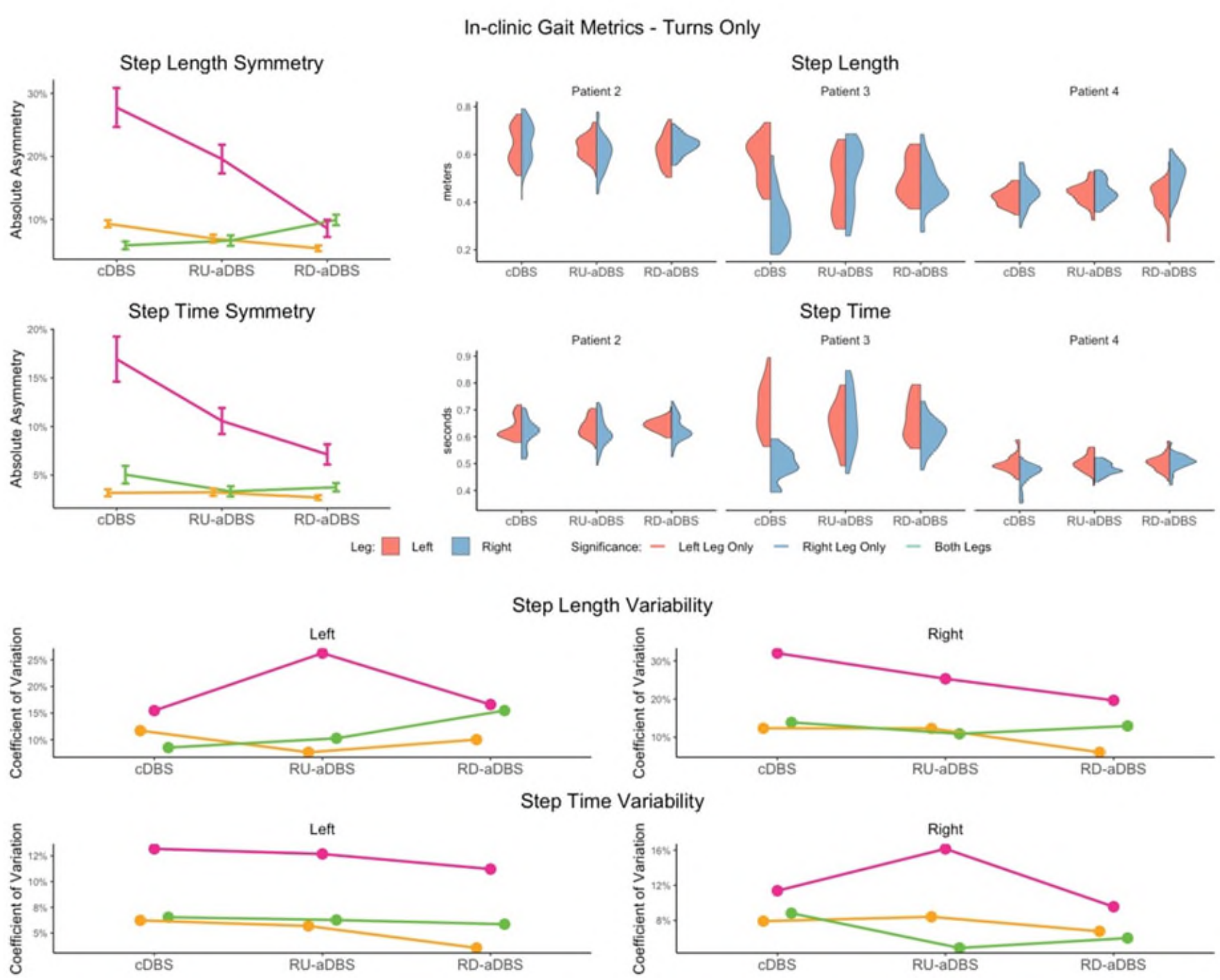

8. **Question:** Why were rump up and rump down chosen? Any individual superiority of one over the other? In particular, ramp up makes sense in patients without side effects of DBS while the opposite in patients with e.g. cortico-spinal spreading of the VTA.

Response: We thank the reviewer for this discussion regarding the volume of tissue activated. We selected these two opposing strategies to investigate contrasting physiological hypotheses: whether providing a phasic prokinetic boost (ramp-up) or a phasic withdrawal of stimulation (ramp-down) is more beneficial for gait. We agree with the reviewer's assessment that ramp-down may be theoretically superior in patients where high-amplitude stimulation causes side effects due to corticospinal spread of the VTA. However, our results indicate that the response to these strategies is complex and not solely predicted by clinical amplitude thresholds. For instance, Patient 3 had the lowest clinical stimulation amplitude (suggesting a lower threshold for side effects), yet their kinematic response profile was similar to patients with much higher amplitudes (Patients 2 and 4). This suggests that the optimal strategy depends on a combination of factors, such as lead location and individual biomechanics, rather than amplitude ramping direction alone.

9. **Question:** Linked to previous comment, the prokinetic effect of GPi is less obvious and I'm not sure how ramping up can result in an immediate benefit of step length.

   Response: We thank the reviewer for this mechanistic inquiry. While we agree that full clinical stabilization can be time-dependent, our empirical data demonstrate that gait kinematics can be modulated on a rapid timescale. In this study, we observed immediate changes in step length synchronized to the stimulation ramp. This rapid response is consistent with the role of the GPi as a "brake" on the motor thalamus. We postulate that the timing of the stimulation ramping, as described in our discussion (lines 421-428), provides phasic disruption precisely during the swing phase to facilitate movement. Previously, our group demonstrated that GPi stimulation induces immediate changes in clinical symptoms (Wang et al., 2018). Furthermore, Malekmohammadi et al. (2018) and Mirpour et al. (2025) have reported that GPi stimulation yields immediate prokinetic effects on motor execution.

10. **Question:** Clinical data are presented in a less intuitive way, probably due to the lack of statistical differences among groups. For example, "at the group level, RU-aDBS significantly increased the odds of reporting fewer falls compared to cDBS (odds ratio [OR] = 4.35)" does it mean that the number of falls were significantly less in the aDBS group?

    Response: We thank the reviewer for this opportunity to clarify. The reviewer is correct: the Odds Ratio of 4.35 signifies a statistically significant reduction in fall

frequency in the RU-aDBS group compared to cDBS. We originally phrased this as "odds of reporting" to reflect the specific statistical model used and the self-reported nature of the outcome. However, we agree this phrasing was less intuitive. We have revised the Results text to state the clinical outcome more directly while retaining the statistical notation.

Change: We have updated the text in the results to clarify comparisons are being made with reported values as opposed to objective measures. Line 238-243.

> "At the group level, RU-aDBS significantly reduced the frequency of reported falls compared to cDBS (odds ratio [OR] = 4.35), while RD-aDBS had no significant effect (Fig. 5a). No group-level changes were detected for freezing episodes. In contrast, aDBS was less likely to improve rigidity compared to cDBS (RU-aDBS: OR = 0.114; RD-aDBS: OR = 0.038), and no significant effects emerged for dyskinesia or tremor (Fig. 5b)."

11. **Question:** Were near-falls addressed?

    Response: We thank the reviewer for raising this clinically important point. Currently, the reliable automated detection of near-falls via wearable sensors remains a significant technical challenge in the field. Consequently, our assessment was limited to patient self-reporting. We reviewed the free-response sections of the daily motor diaries, which allowed patients to document adverse events not covered by standard questions, and found that no near-falls were reported during the study period. However, we acknowledge that relying on self-reporting may underestimate the frequency of such events.

    Change: We have added text to describe this limitation of our study. Line 404-407.

    > "Furthermore, our assessment of at-home safety was limited to a self-reported motor diary. Objective quantification of near-falls was not possible because of the lack of validated automated detection standards for these events."

12. **Question:** "No group-level changes were detected for freezing episodes": how about wearable data? Overall this points to a less impactful effect of aDBS. To this point, Authors' claims in the discussion about the efficacy might not be concordant with Results.

Response: We thank you for this discussion. The wearable data captured by the Rover system (ankle-worn accelerometers) out of the clinic cannot detect falls. We have edited the discussion to better reflect the efficacy we observed towards freezing and to point out that we think aDBS functions primarily to enhance gait stability rather than prevent freezing.

Change: We have edited the discussion to better reflect the impact of aDBS on falls and freezing. Line 392-395.

> "Crucially, while aDBS appeared to reduce fall frequency, we did not observe the same reduction in freezing episodes. This distinction highlights the value of continuous monitoring in dissecting specific symptom responses, suggesting that aDBS may primarily enhance gait stability rather than preventing freezing initiation."

13. **Question:** "Odds of improvement in rigidity were low under both strategies (RUaDBS: OR = 0.114; RD-aDBS: OR = 0.038)": evaluated how? At home?

    Response: We thank the reviewer for this inquiry regarding the assessment methodology. To answer the question directly: Yes, rigidity was evaluated at home via patient self-reporting. Patients rated the severity of their rigidity daily using the home motor diaries. As noted in our response to Comment 10, we have revised the Results section to explicitly state that these outcomes are "reported" values, ensuring the reader distinguishes these subjective ratings from clinician-administered objective exams.

14. **Question:** Videos of patients would be of interested.

    Response: We thank the reviewer for their curiosity. We have added a new supplementary video (Supplementary Information Video 4) showing Patient 2 on cDBS and RU-aDBS. Videos are taken from our IMU based motion capturing system (Xsens) to protect our patient's identity.

    Change: We edited the text in the results to refer to this new video. Line 229.

    > "...against each patient's cDBS setting (Supplementary Video 4)."

15. **Question:** Authors have recently published papers from the same cohort, how are these studies blending with each other?

Response: We thank the reviewer for following our work on this cohort. These studies represent two distinct, complementary approaches of adaptive control for gait dysfunction. The current manuscript focuses on a modulating stimulation within the gait cycle (milliseconds). The other study focuses on using a data-driven approach to identify a continuous DBS parameter that is optimized for gait (published study), with the ultimate goal of identifying walking vs all non-walking states using LFP biomarkers and use that control signal to switch to this gait-optimized setting when someone is walking (in progress). These approaches are not mutually exclusive but hierarchical. We envision a future "blended" therapy where the system first detects the walking state and then engages the phase-dependent modulation to restore dynamic gait control. This manuscript establishes the feasibility of the phase-dependent modulation.

16. **Question:** What's the role and effect of L-dopa?

    Response: We thank the reviewer for this important question regarding the interaction between medication state and neural dynamics. Consistent with established literature, we observed that L-dopa tends to "normalize" the neural signal. In a representative dataset from Patient 2, where we successfully captured overground walking in both OFF and ON medication states prior to DBS activation, L-dopa resulted in a reduction in gait variability and more consistent gait event timing (see attached figure below, colored markers). Additionally, the gait-phase-related neural modulation became more distinct. L-dopa appeared to regularize the physiological modulation during the contralateral swing phase. This observation reinforces our methodological decision to include "LOW" medication states in our biomarker search. By training the algorithm on data where the modulation might be weaker (LOW/end-of-dose), we ensured the detector remained robust even when the "clarifying" effect of L-dopa was diminished. While a comprehensive pharmacological analysis is reserved for future study, these preliminary data suggest that L-dopa and aDBS may work synergistically to restore physiological gait-phase modulation.

Redacted

17. **Question:** “Coordinated reset stimulation, which is designed to desynchronize pathological synchrony, requires precise temporal delivery and has been associated with motor improvements”: overall most DBS experts are skeptical that this is effective.

    Response: We appreciate the reviewer’s perspective on this literature. We agree that the clinical efficacy of coordinate reset remains controversial and evidence for its translation to humans is limited. To avoid conflating our findings with this debate, we have removed the references to coordinated reset. Instead, we have refined the discussion to focus specifically on the concept of network entropy and the hypothesis of restoring a functional equilibrium, as supported by the cited literature regarding pathological synchrony vs. hyperkinetic states.

    Change: We have deleted the text referencing Coordinated Reset. The paragraph has been revised to focus on modulating network entropy toward a functional equilibrium. Lines 379-386.

    > “The timing of stimulation may be key to modulating network dynamics. Current evidence suggests that pathological states can occur at different ends of network entropy. High entropy (i.e., desynchronized activity) in basal ganglia activity may worsen parkinsonism, whereas excessively low entropy

is associated with hyperkinetic disorders. This implies that effective neuromodulation requires pushing the network between these extremes. We hypothesize that gait-phase synchronized aDBS introduces semi-random stimulation variability, modulating network entropy towards a functional equilibrium that supports effective gait control without destabilizing other motor functions."

18. **Question:** "Patient 1, who exhibited severe dyskinesia, showed no improvements in gait metrics, possibly due to the chronic instability of stimulation settings and limited acute testing duration": this is mentioned for the first time in the discussion

    Response: We thank the reviewer for this attention to detail. We have edited the text in the result to bring up the unique clinical profile of this patient earlier.

    Change: We have added the following text. Line 211-214.

    > "Notably, this lack of response corresponded to a distinct clinical profile. Patient 1 experienced chronic instability of effective cDBS settings, manifesting as severe dyskinesia, and required the longest duration of standard clinical optimization (520 days).

19. **Question:** What's the meaning of "-/-" in table 1?

    Response: We thank the reviewer for identifying this lack of clarity. The symbol "-/-" indicates data that could not be collected due to specific logistical or safety constraints. For Patient 1, recruitment occurred during peak COVID-19 restrictions (2021). The screening was conducted via telemedicine, which means the physical assessment of the rigidity and postural stability components of the MDS-UPDRS III could not be captured. For Patient 5, the patient requires a walker for daily walking. Because the Mini-BESTest requires unassisted standing and stepping, the assessment was contraindicated due to high fall risk.

    Change: We have added the following footnote to Table 1:

    > "Note: '-/-' indicates data not acquired due to remote assessment restrictions (Patient 1) or safety contraindications (Patient 5)."

20. **Question:** Why was LOW used? This is 'low medication state defined as the same period between the end of their current medication cycle to the next'. It seems quite convoluted to me, was it the 'off' for all conditions?

Response: We appreciate the opportunity to clarify this definition and the rationale behind the study design. To answer the question directly: No, the "LOW" condition did not serve as a standard "OFF-medication" control for the efficacy analysis. We specifically captured data in the LOW-medication state to characterize the neural activity across different medication states. This was critical for our biomarker discovery process. By including both ON and LOW states in our data-driven search, we ensured that the selected gait-phase biomarker was robust to medication fluctuations and would function reliably as the patient experienced "wearing-off" effects throughout the day. Additionally, we did not utilize a clinically defined "OFF" state as a comparator because, during pre-DBS-activation assessments, several patients experienced severe bradykinesia and rigidity, making them unable to perform the walking tasks safely.

We thank the editors for the opportunity to revise and resubmit our manuscript, and we are grateful for the thoughtful and constructive feedback from the reviewers. We have substantially revised the manuscript to address all comments, clarify the conceptual framework, and strengthen both the technical and clinical interpretation of our findings. In particular, we have clarified the conceptual positioning of this work as introducing a behavior-contingent, movement-phase synchronized adaptive DBS paradigm, distinct from existing beta-state driven adaptive DBS approaches.

Below we provide a detailed, point-by-point response to each comment.

Reviewer #1

Comment:
The manuscript has improved substantially, and prior concerns regarding methods and limitations have been addressed.

Response:
We thank the reviewer for their careful reassessment and for recognizing the improvements made in the revised manuscript. We have further refined the manuscript to improve clarity around feasibility and scalability, explicitly framing this study as a translational proof-of-concept and outlining future directions for streamlining biomarker identification in larger cohorts.

Reviewer #2

1. “While less common than in the STN, this effect is well-documented in the GPi, particularly with ventral stimulation”: although possible, ventral GPI is mainly anti-kinetic, the dorsal stimulation of the nucleus can cause dyskinesias. In any case, these are rare occurrences and almost never disabling

   Response: We thank the reviewer for this important clarification and agree that stimulation-induced dyskinesia is less common in GPi compared to STN. Our intent is not to emphasize the frequency or severity of dyskinesia per se, but to highlight a broader principle: continuous DBS, whether in STN or GPi, may produce suboptimal or adverse motor effects when stimulation does not adapt to fluctuations in behavioral or medication state.

   We further clarify that one potential mechanism underlying these effects is network-level entrainment, whereby continuous stimulation imposes a temporally invariant neural state, potentially limiting physiological flexibility of cortico-basal ganglia circuits (Olaru et al., 2024, 2025).

Change: We have changed the text in the introduction to more clearly convey this point of stimulation-induced entrainment.

Lines 59-68:
"While most of these electrophysiological insights derive from the subthalamic nucleus (STN), parallel dynamics exist in the internal globus pallidus (GPi). **Without accounting for these fluctuating neural dynamics, continuous stimulation of the STN or GPi may lead to suboptimal or adverse motor effect. For instance, during "on" medication states, when dopaminergic therapy effectively controls motor symptoms, continuous DBS can cause stimulation-induced dyskinesia[28,31-34]. From a network perspective, although neural entrainment to stimulation is often associated with improved motor performance, continuous stimulation may impose a persistently entrained, temporally invariant neural state[29,35,36], limiting the capacity of cortico-basal ganglia circuits to dynamically reconfigure in accordance with ongoing behavioral demands.**"

2. "We have updated the text in the results to clarify comparisons are being made with reported values as opposed to objective measures. Line 238-243": reporting clinical effects in terms of ORs remains odd.

   Response:
   We thank the reviewer for this point. All motor symptoms were rated by the patient using ordinal categories, and therefore, analyzed using an ordinal statistical model and reported as odds ratios. The model treated categories as ordered outcomes from worst to best for each symptom, meaning the statistics for number of falls and number of freezing episodes started at 5+ and ended at 0. In this context, an odds ratio of 4.35 indicates that participants were significantly more likely to report lower fall-frequency categories under RU-aDBS compared to cDBS, consistent with a reduction in fall frequency. We have updated the text in the results and added additional information in the statistical analysis section in the methods.

   Change

   Lines 240-246:
   "At the group level, RU-aDBS was associated with a significant reduction in reported fall frequency compared to cDBS (odds ratio [OR] = 4.35**), indicating a higher likelihood of patients reporting lower fall-frequency categories** (Fig. 5a). RD-aDBS had no significant effect. No group-level changes were observed for freezing episodes. In contrast, patients were less likely to report improved rigidity on aDBS compared to cDBS (RU-aDBS: OR = 0.114; RD-aDBS: OR = 0.038), and no significant effects were observed for dyskinesia or tremor (Fig. 5b)."

   Lines 981-987:
   "Ratings for the cardinal symptoms were recorded on a 3-point ordinal scale: "Worse," "Same," or "Better." For the number of falls and number of freezing episodes, patients selected one of four categories: 0, 1, 2-4, or 5+. To evaluate whether stimulation condition

influenced symptom perception, the number of falls, or number of freezing episodes, a cumulative link model was used to assess the probability of reporting a change from cDBS across conditions. The category order was defined as follows: “Worse,” “Same,” and “Better” for rigidity, bradykinesia, and tremor; and 5+, 2–4, 1, and 0 for the number of falls and freezing episodes.”

3. “rigidity was evaluated at home via patient self-reporting”: no PD experts would agree that this is feasible, as rigidity is by definition assessed by the examiner’s act of moving a body part. In fact, very rarely a PD patient would complain of ‘rigidity’ or ‘stiffness’.

   Response:

   We thank the reviewer for this important point and agree that rigidity, as a clinical construct, is optimally assessed through examiner-based evaluation. In this study, patient-reported measures were intended to capture subjective perceptions of stiffness rather than clinically assessed rigidity. Participants were trained in standardized motor diary reporting and routinely documented fluctuations in motor state, including perceived stiffness, as part of established clinical practice.

   We have revised the manuscript to clarify that patient-reported rigidity reflects subjective perception of stiffness rather than clinically measured rigidity. To avoid misinterpretation, we have: 1) explicitly relabeled this measure as “patient-reported stiffness,” and 2) clarified that formal rigidity assessments were obtained during in-clinic examinations using standardized scales (MDS-UPDRS III).

   We further emphasize that the primary clinical focus of this study is gait-related outcomes, which were assessed using objective kinematic measures and self-reported fall frequency. These endpoints remain unaffected by this limitation.

   These revisions improve clarity and align terminology with accepted clinical standards.

   Changes (lines – multiple)
   Instances of the text “rigidity” was replaced with “patient-reported stiffness” or “perception of stiffness” when related to the motor diary report and analysis.

   Figure (#5)
   The text “Rigidity” has been changed to “Patient-reported Stiffness,” with the figure caption text changed to match.

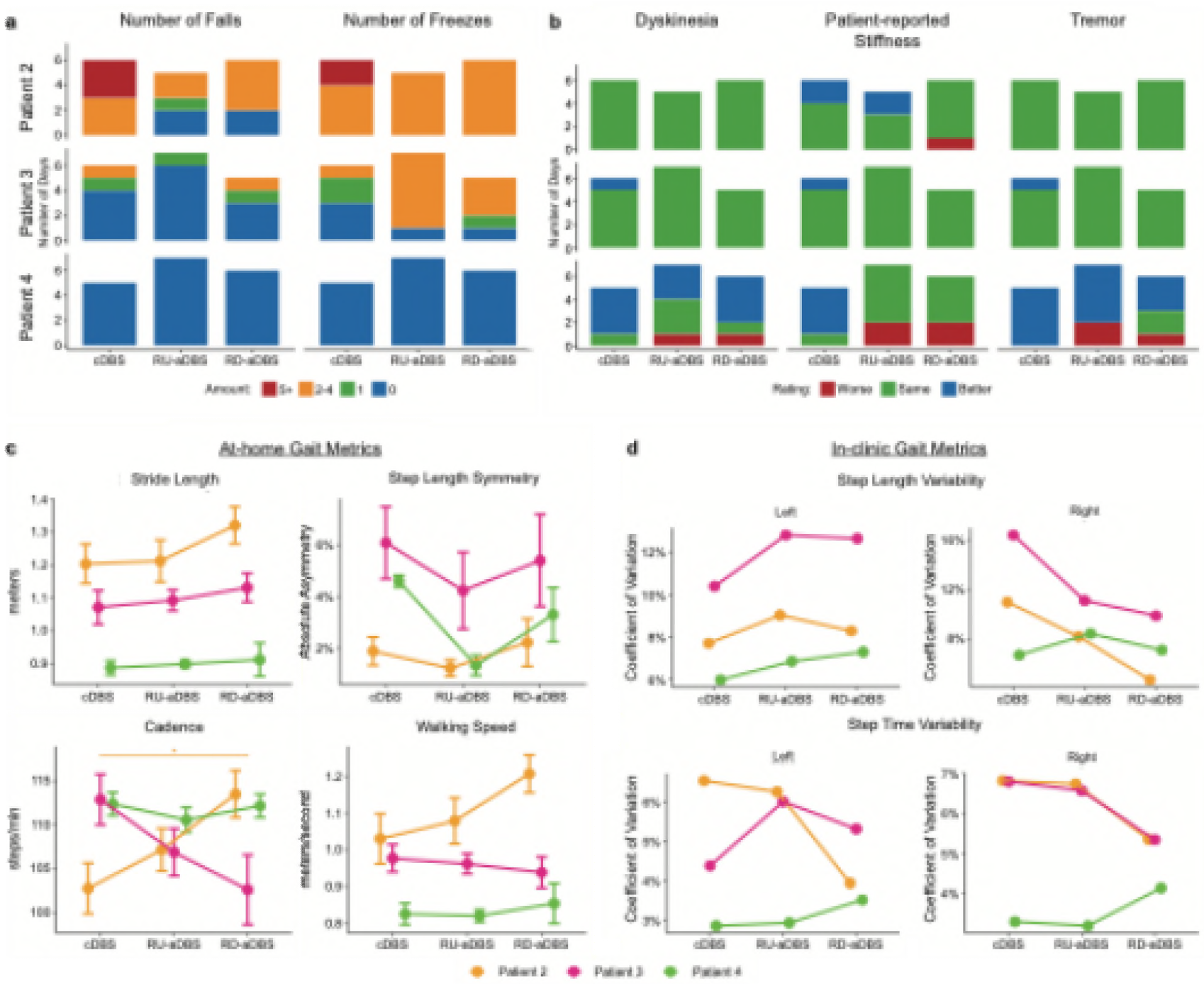

4. “Videos are taken from our IMU based motion capturing system (Xsens) to protect our patient’s identity”: this is odd and in contrast with the vast majority of clinical studies, where real videos are presented.

Response:

We thank the reviewer for this comment. Motion capture–based visualization (Xsens) was used to 1) ensure patient privacy and comply with institutional guidelines, and 2) to provide unobstructed visualization of gait during recordings in which a researcher was physically guarding the patient for safety.

We agree that direct video visualization is valuable. Accordingly, we have updated the supplementary materials to include synchronized camera footage, with facial features blurred when feasible, to complement the motion capture data.

Change:

Supplementary Videos 2 and 4 have been updated to include synchronized camera footage of the patient, with facial features blurred to preserve privacy. For Video 4, the camera was positioned differently from the Xsens recording system; as a result, the overlaid animation and camera footage show the subject walking in opposite directions. We have clarified this in the video legend to avoid confusion.

Camera footage was not added to Supplementary Video 1 because a researcher who was guarding the patient partially obscured the patient throughout the recording. In addition, as

this video is intended to highlight rapid stimulation state transitions during the gait cycle, which is more clearly visualized using the slowed Xsens-derived animation, and including partially obstructed camera footage would detract from its clarity.

Supplementary Video 3 (dyskinesia) does not include camera footage because the dyskinetic episode occurred before video recording was initiated. However, the purpose of this video is to demonstrate that non-gait leg movements do not spuriously trigger state changes. The Xsens-derived animation clearly illustrated the dyskinetic leg movements.

5. Overall, I still don't grasp whether this new model of stimulation is superior to the already approved aDBS merely based on pallidal beta.

Response:
We thank the reviewer for highlighting this important conceptual point. We agree that the original framing may have inadvertently suggested a direct comparison with beta-based adaptive DBS, and we have substantially revised the manuscript to clarify this distinction.

This study does not aim to demonstrate superiority over existing beta-based aDBS. Rather, it introduces and validates a fundamentally different adaptive control paradigm. Beta-based aDBS operates on tonic neural signals that reflect global motor or medication state and is well suited for continuous symptoms such as bradykinesia or rigidity. In contrast, the present approach is behavior-contingent and operates on sub-second timescales, synchronizing stimulation to discrete phases of a dynamic motor behavior.

Gait represents a temporally structured, cyclical behavior requiring rapid coordination across distributed neural circuits. As such, control strategies based on slow-varying or tonic signals are not well matched to the temporal demands of gait. The approach presented here directly targets this gap by linking stimulation to movement phase itself.

We have revised the Introduction and Discussion to explicitly distinguish:
(i) beta-based, state-driven adaptive control and
(ii) behavior-contingent, phase-locked control
and to clarify that these approaches are complementary rather than competing control strategies.

We further emphasize that gait dysfunction remains poorly addressed by current DBS paradigms, including beta-based aDBS, despite adequate control of other motor symptoms. The clinical improvements observed here, particularly in gait variability, symmetry, and falls, support the translational relevance of this distinct control strategy.

We believe this clarification addresses the concern and more accurately positions the contribution of this work.

We have edited the text accordingly:

Introduction

Lines 69-81:

"Adaptive DBS (aDBS), which adjusts stimulation parameters based on real-time neural signals, has emerged as a promising approach to address changes in physiological demand[44,45]. Most current aDBS implementations rely on relatively slow-varying, tonic neural features, most commonly beta-band activity, that reflect global motor or medication state. These state-adaptive strategies are effective for improving "on" times without bothersome dyskinesia[46–50] but are not inherently aligned with the rapid temporal structure of dynamic motor behaviors such as gait.

Here, we introduce a behavior-contingent adaptive DBS paradigm that operates on a fundamentally different principle. Rather than modulating stimulation based on global motor state, this approach synchronizes stimulation to discrete phases of the gait cycle, enabling sub-second, movement-phase-locked modulation of neural activity (Fig. 1a). By aligning stimulation delivery with the temporal structure of locomotion, this strategy is designed to complement existing state-adaptive approaches and specifically target dynamic motor impairments."

Discussion

Lines 296-300:

"Improving advanced gait disturbances with traditional DBS has remained a major clinical challenge. Here, we developed and implemented a novel gait-phase synchronized aDBS therapy that rapidly modulates stimulation amplitude during contralateral leg swing on a sub-second time scale**: a behavior-contingent adaptive DBS paradigm that differs fundamentally from existing approaches.**"

...

Lines 307-314:

"In this study, we introduce a gait-phase synchronized aDBS paradigm that achieves sub-second, movement-locked modulation of stimulation amplitude, driven by individualized neural biomarkers derived from cortical–pallidal circuits. This represents a significant technical advance over prior aDBS approaches, which have largely operated on slower time scales (seconds to minutes)[46,55] and targeted canonical beta activity[47–49,56–58] without consideration of the movement phase. **Furthermore, this approach introduces a complementary control framework that operates on the timescale of behavior itself rather than as a direct alternative to existing state-based (e.g., beta-driven) adaptive DBS strategies.**"

…

Lines 418-424:

"Our results demonstrate that sub-second, gait-phase synchronized adaptive DBS—driven by individualized cortical–pallidal biomarkers—can restore dynamic gait control in Parkinson's disease while preserving broader motor function. By targeting stimulation to the precise temporal windows when pathological activity is most disruptive, this approach overcomes key limitations of continuous DBS, **extends adaptive neuromodulation beyond conventional beta state-based paradigms**, and reveals mechanistic insights into the circuit-level control of locomotion."

Abstract

Lines 21-38:

"Gait dysfunction in Parkinson's disease (PD) is a major source of disability and is often insufficiently treated by continuous deep brain stimulation (cDBS). **While adaptive DBS (aDBS) has shown efficacy for other motor symptoms using beta-based, state-driven neural signals, gait is a dynamic, cyclical behavior that may require temporally precise modulation. Here, we evaluated a behavior-contingent aDBS approach that synchronizes stimulation to gait phase.** We report a single-center, blinded, randomized, cross-over study evaluating the feasibility of identifying patient-specific biomarkers to drive aDBS. The primary outcome was feasibility of successful identification of gait phase biomarkers to implement aDBS. Five PD participants undergoing pallidal DBS and subdural electrode paddle implantation were enrolled. We successfully identified personalized gait-phase biomarkers from cortical or pallidal field potentials in all five patients and embedded them into a bidirectional neurostimulator. During acute in-clinic testing, aDBS improved step variability and step symmetry versus cDBS. Three participants subsequently completed a double-blinded, multi-day crossover phase. In this setting, aDBS maintained general motor symptom control, reduced falls, and yielded patient-specific gait improvements. No adverse events occurred and aDBS was well tolerated. These findings establish the feasibility of biomarker-driven, movement-synchronized neuromodulation and support the development of a larger randomized trial to determine clinical efficacy. Clinical trial registration: NCT04675398."
